# Neuron-secreted NLGN3 ameliorates ischemic brain injury via activating Gαi1/3-Akt signaling

**DOI:** 10.1038/s41419-023-06219-8

**Published:** 2023-10-25

**Authors:** Zhi-guo Chen, Xin Shi, Xian-xian Zhang, Fang-Fang Yang, Ke-ran Li, Qi Fang, Cong Cao, Xiong-hui Chen, Ya Peng

**Affiliations:** 1 Department of Neurology, The First Affiliated Hospital of Soochow University, Suzhou, China; 2 Department of Neurology and Clinical Research Center of Neurological Disease, The Second Affiliated Hospital of Soochow University, Institute of Neuroscience, Soochow University, Suzhou, China; 3 Department of Neurology, Affiliated Hospital 6 of Nantong University, Yancheng Third People’s Hospital, Yancheng, China; 4 Department of Emergency Surgery, First Affiliated Hospital of Soochow University, Suzhou, China; 5 Department of Neurosurgery, The Third Affiliated Hospital of Soochow University, Changzhou, China

**Keywords:** Cell death in the nervous system, Stroke

## Abstract

We here tested the potential activity and the underlying mechanisms of neuroligin-3 (NLGN3) against ischemia-reperfusion-induced neuronal cell injury. In SH-SY5Y neuronal cells and primary murine cortical neurons, NLGN3 activated Akt-mTOR and Erk signalings, and inhibited oxygen and glucose deprivation (OGD)/re-oxygenation (OGD/R)-induced cytotoxicity. Akt activation was required for NLGN3-induced neuroprotection. Gαi1/3 mediated NLGN3-induced downstream signaling activation. NLGN3-induced Akt-S6K1 activation was largely inhibited by Gαi1/3 silencing or knockout. Significantly, NLGN3-induced neuroprotection against OGD/R was almost abolished by Gαi1/3 silencing or knockout. In vivo, the middle cerebral artery occlusion (MCAO) procedure induced NLGN3 cleavage and secretion, and increased its expression and Akt activation in mouse brain tissues. ADAM10 (A Disintegrin and Metalloproteinase 10) inhibition blocked MCAO-induced NLGN3 cleavage and secretion, exacerbating ischemic brain injury in mice. Neuronal silencing of NLGN3 or Gαi1/3 in mice also inhibited Akt activation and intensified MCAO-induced ischemic brain injury. Conversely, neuronal overexpression of NLGN3 increased Akt activation and alleviated MCAO-induced ischemic brain injury. Together, NLGN3 activates Gαi1/3-Akt signaling to protect neuronal cells from ischemia-reperfusion injury.

## Introduction

Ischemic stroke is a leading cause of human morbidities and mortalities around the world [1, 2]. It affects over 1.5 million people globally each year [3]. For clinical management of acute ischemic stroke, the treatment guidelines recommend early thrombolysis. Yet the clinical efficiency is largely time-dependent [4, 5]. The incidence of ischemic stroke, however, has been rising in recent years [4, 5]. It is thereby important to further explore novel and more effective treatment strategies [5–7]. Ischemia-reperfusion will cause robust reactive oxygen species (ROS) production and oxidative injury to affected neurons [8, 9]. Oxygen and glucose deprivation (OGD)/re-oxygenation (OGD/R) [10] procedure mimics ischemia-reperfusion injuries to cultured neurons (and neuronal cells) [11–14].

Neuroligin-3 (NLGN3) is a primary family member of NLGN family proteins [15, 16]. It is a type I membrane protein and a cell adhesion molecule, locating at the neuron’s postsynaptic membrane [17]. It can bind to β-neurexin, which helps form synapses between neurons [18, 19]. NLGN3 is a synaptic protein that could be cleaved and secreted in an activity-dependent manner [18–21]. It is recognized as a key neuronal-derived factor promoting neuron activity-dependent glioma growth [18–20].

NLGN3 is cleaved by ADAM10 (A Disintegrin and Metalloproteinase 10) and is secreted from neurons, inducing phosphorylation and activation of several key receptor tyrosine kinases (RTKs) and downstream signaling cascades in glioma cells [18–21]. Ligand-activated RTKs, including the neurotrophic factor receptors (TrkB, TrkA GDNFR *etc*), epidermal growth factor receptor (EGFR), fibroblast growth factor receptor (FGFR), and platelet-derived growth factor receptor (PDGFR), are capable of promoting neuronal survival through activating downstream PI3K-Akt and other pro-survival cascades [12, 22–28]. Many studies have also focused on the neuroprotective potential of non-ligand agents that can activate RTKs and can elicit effects similar to those of the ligands [12, 22–28]. We here tested the potential activity and underlying mechanisms of NLGN3 against ischemia-reperfusion-induced neuronal cell death.

The inhibitory α subunits of G proteins (heterotrimeric guanine nucleotide-binding proteins), or Gαi proteins, have three subunits, including Gαi1, Gαi2, and Gαi3 [29]. Gαi proteins binding to GPCRs (G protein-coupled receptors) can inhibit adenylate cyclase (AC) and cyclic AMP (cAMP) production [29]. Alternatively, our previous studies have shown that Gαi1 and Gαi3 proteins are vital in mediating signal transduction of several RTKs, including EGFR [30], FGFR1 [31], keratinocyte growth factor receptor (KGFR) [32], brain-derived neurotrophic factor (BDNF) receptor TrkB [33], and vascular endothelial growth factor receptor 2 (VEGFR2) [34]. We here found that Gαi1 and Gαi3 mediated NLGN3-induced Akt activation and neuroprotection against ischemia-reperfusion injury.

## Materials and methods

### Reagents

NLGN3, puromycin, polybrene, U0126, GI254023X, and MK-2206 were purchased from Sigma-Aldrich (St. Louis, Mo). Fetal bovine serum (FBS), RPMI, DMEM, antibiotics, and other cell culturing reagents were purchased from Gibco Co. (Shanghai, China). Antibodies for adenine nucleotide translocase 1 (ANT1, ab102032) and cyclophilin-D (CypD, sc-137136) were reported early [35]. Other antibodies utilized in this study were described in our previous studies [31, 36–39]. The primers, sequences, and viral constructs were obtained from Genechem Co. (Shanghai, China), unless otherwise mentioned.

### Cell culture

SH-SY5Y neuronal cells were from Dr. Liu at Jiangsu University [40]. SH-SY5Y were differentiated and cultured as described [10, 40]. The isolation and primary culture of murine cortical neurons were performed by the described protocols [40–42]. At day-10 (DIV), over 98% of cells were primary murine cortical neurons. The protocols of the present study were approved by the Ethics Committee and Institutional Animal Care and Use Committee (IACUC) of Soochow University.

### OGD/re-oxygenation

The OGD/R procedure was described previously [11, 41]. In brief, for OGD/R stimulation, SH-SY5Y cells or cortical neurons were first cultured in glucose-free DMEM in an incubator (Heraeus, Hanau, Germany) containing 0.5% O_2_, 94.5% N_2_ and 5% CO_2_ for 4 h at 37 °C (OGD). Afterwards, SH-SY5Y cells or neurons were grown in complete medium with serum in 95% air and 5% CO_2_ at 37 °C (OGD/R) for designated time periods. Neuronal cells in the norm-oxygenated DMEM containing glucose were labeled as “Mock” control cells.

### Cell Counting Kit-8 and cell death assays

SH-SY5Y neuronal cells or primary murine cortical neurons were plated into poly-L-lysine-coated 96-well plates (at 3 ×10^4^ cells/cm^2^). Following the designated treatment, cell viability was tested through the Cell Counting Kit-8 (CCK-8) kit (Dojindo Molecular Technologies, Gaithersburg, MD). CCK-8 optical density (OD) in each well was recorded at 550 nm. Cell death was tested by Trypan blue staining using an automatic cell counter.

### Lactate dehydrogenase assay

Cell necrosis was quantified by measuring lactate dehydrogenase (LDH) releasing to the medium. In brief, LDH activities in cell lysates and supernatants were separately measured by a LDH assay kit (Sigma), with the LDH absorbance tested at 490 nm. The % LDH release from the cells, indicating cell necrosis intensity, was calculated by dividing absorbance of medium LDH absorbance to the total (medium plus lysates) LDH absorbance [40, 43].

### Caspase-3/-9 activity

Following treatment, 20 μg of protein lysates per treatment were dissolved in the caspase assay buffer containing 7-amino-4-trifluoromethylcoumarin (AFC)-conjugated caspase-3/-9 substrates. The Infinite 200 PRO reader was utilized to examine AFC activity at 400 nm excitation and 505 nm emission after 3 h incubation.

### Terminal deoxynucleotidyl transferase dUTP nick end labeling staining

SH-SY5Y neuronal cells or primary murine cortical neurons were plated into poly-L-lysine-coated 96-well plates (at 3 × 10^4^ cells/cm^2^). Following the designated treatment, the terminal deoxynucleotidyl transferase dUTP nick end labeling (TUNEL) In Situ Cell Death Detection Kit (Roche Diagnostics Co., Shanghai, China) was utilized to quantify cell apoptosis. In brief, cell nuclei were co-stained with TUNEL and DAPI, and visualized under a fluorescent microscope (Leica). At least 1, 200 cell nuclei in five random views (1 × 200 magnification) were counted to calculate the average TUNEL ratio (% vs. DAPI).

### Annexin V-FACS assay of apoptosis

Following treatment, neuronal cells were washed, re-suspended and co-stained with Annexin V and propidium Iodide (PI). A FACS machine (BD, Shanghai, China) was utilized to sort the cells, with Annexin V ratio recorded.

### Single-strand DNA detection

Neuronal cells were initially seeded into the poly-L-lysine-coated six-well plates at 120, 000 cells per well. Following treatment, the single-strand DNA (ssDNA) apoptosis ELISA kit (Merck Millipore, Shanghai, China) was utilized to quantify DNA break intensity according to the attached protocol, with ELISA OD examined at 405 nm for each well.

### Mitochondrial depolarization

With mitochondrial membrane potential collapse and depolarization, JC-1 shall form monomers in mitochondria, emitting green fluorescence [44]. Following treatment, neuronal cells were stained with the JC-1 (10 μg/mL) dye, washed and its green intensity examined under a fluorescence spectrofluorometer (at 525 nm, Hitachi, Japan). The JC-1 fluorescence images, integrating both the green (at 525 nm) and red (at 675 nm) fluorescence channels, were presented.

### Western blotting

The detailed protocols of Western blotting were extensively described in the previous studies [30, 33]. The exact same amount of quantified protein lysates (30–40 μg) per each lane was loaded. When necessary, the same set lysates were run in parallel gels. The ImageJ software was utilized to quantify the band intensity. The uncropped blotting images were presented in Fig. S2.

### Mitochondrial immunoprecipitation

The mitochondrial immunoprecipitation assays were carried out using the described protocol [11, 41, 45, 46]. Briefly, the mitochondrial fraction lysates of SH-SY5Y cells were obtained, pre-cleared, and incubated with the anti-CypD antibody [35]) overnight. The protein IgG “beads” (Sigma) were then added to extract the CypD-bound mitochondrial immune complexes. CypD-p53-ANT1 association was then tested by Western blotting assays.

### Lipid peroxidation assays

Neuronal cells were seeded into the poly-L-lysine-coated six-well plates at 120,000 cells per well. Following the designated treatment, the thiobarbituric acid reactive substances (TBAR) activity was examined to reflect the cellular lipid peroxidation intensity, using the described protocol [42, 47].

### ROS assays

Neuron cells were initially seeded into the poly-L-lysine-coated six-well plates at 120,000 cells per well. Following the applied treatment, cells were stained with CellROX (25 μg/mL), washed, and CellROX red fluorescence intensity examined under a spectrofluorometer (F-7000, Hitachi, Japan) at 625 nm. CellROX fluorescence images were presented as well.

### Constitutively-active mutant Akt1

Neuron cells were initially seeded into the poly-L-lysine-coated six-well plates at 120, 000 cells per well and were infected with the constitutively-active Akt1 (caAkt1, S473D)-expressing lentivirus. After 12 h, puromycin (2.0 μg/mL) was utilized to select stable cells for another 48 h. The caAkt1 expression in the stable cells was verified by Western blotting assays.

### Akt1/2 shRNA

Neuron cells were initially seeded into the poly-L-lysine-coated six-well plates at 120,000 cells per well and were transfected with Akt1/2 shRNA lentiviral particles (sc-43609-V, Santa Cruz Biotech, Santa Cruz, CA). After 24 h, puromycin (2.0 μg/mL) was utilized to select stable cells for another 72 h. Akt1/2 silencing was verified by Western blotting assays.

### Gαi1/3 shRNA

SH-SY5Y neuronal cells were seeded into six-well plates at 60% confluence and treated with the Gαi1 shRNA lentiviral particles plus the Gαi3 shRNA lentiviral particles [34]. After 24 h the stable cells were selected by puromycin for another 72 h. Gαi1/3 double silencing was verified by Western blotting assays. The scramble control non-sense shRNA (“shC”) was tranduced to SH-SY5Y cells as control.

### CRISPR/Cas9-induced Gαi1/3 double knockout

SH-SY5Y neuronal cells at 50–60% confluence were transfected with Cas9-expressing construct (Genechem) via Lipofectamine 3000 (Invitrogen). Next, the lenti-CRISPR/Cas-9 Gαi1 KO construct and the lenti-CRISPR/Cas-9 Gαi3 KO construct, as described in our previous studies [34, 36], were co-transduced to the Cas9-expressing SH-SY5Y cells. Stable cells were established by puromycin selection (for additional 96 h). Gαi1/3 double KO (DKO) was screened in the stable cells. The lenti-CRISPR/Cas-9 empty vector with non-sense sgRNA (“Cas9-C”) was transduced to control cells.

### Gαi1/3 siRNA

The siRNA targeting the murine Gαi1 and the siRNA targeting the murine Gαi3 were designed, synthesized, and verified by Genechem (Shanghai, China). The cultured primary murine cortical neurons were transfected with 200 nM of the Gαi1 siRNA plus the Gαi3 siRNA or the scramble control siRNA (“siC”) by Lipofectamine 3000 (Invitrogen) for 48 h. Expression of Gαi1 and Gαi3 was tested by Western blotting assays.

### Genetic modifications in vivo

The mNLGN3 shRNA sequence, the Gαi1/3 shRNA sequence [33, 38], or the mNLGN3 cDNA sequence was inserted into *Eco*RI and *Bam*HI sites of the GV680 AAV8 vector [48]. The vector along with the viral packaging plasmids were co-transfected into HEK293 cells, producing recombinant adenovirus, which were then filtered, enriched, and quantified.

### The middle cerebral artery occlusion model and 2,3,5-triphenyltetrazolium hydrochloride staining

The detailed protocols for middle cerebral artery occlusion (MCAO), 2,3,5-triphenyltetrazolium hydrochloride (TTC) staining, and data quantification were described in our previous study [49]. The procedures of immunofluorescence in the brain sections were also reported early [49]. The animal protocols were conducted in according to the Institutional Animal Care and Use Committee and the Ethic Committee of Soochow University.

### Behavior tests

The detailed protocols of neurological (Garcia) scores (performed 24 hours after MCAO), the foot-fault test (performed 14 days after MCAO) and data quantification were described in detail in our previous study [49].

### Statistical analysis

Data were all with normal distribution and were expressed as mean ± standard deviation (SD). The statistical difference between multiple groups was performed by one-way ANOVA with Scheffe’s test (SPSS23.0, Chicago, CA). The significance between two treatment groups was analyzed by a two-tailed unpaired *t* test (Excel 2007). *P*-values < 0.05 were considered as statistically significant. The in vitro experiments were repeated at least five times and consistent results were obtained.

## Results

### NLGN3 ameliorates OGD/R-induced neuronal cell death

**Fig. 1:**
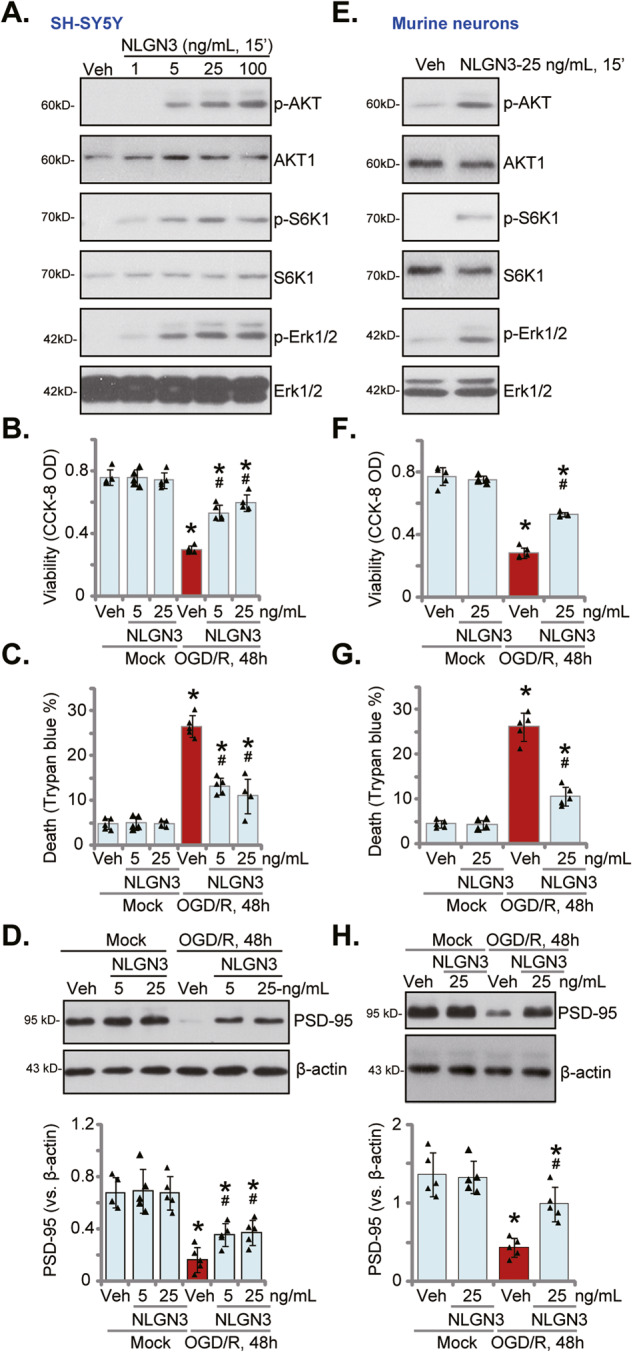
NLGN3 ameliorates OGD/R-induced neuronal cell death. The differentiated SH-SY5Y neuronal cells or the primary murine cortical neurons were treated with NLGN3 (at applied concentrations) or vehicle control (PBS, “Veh”) for 15 min, expression of listed proteins was tested by Western blotting assays (**A**, **E**). SH-SY5Y cells or the primary murine cortical neurons were pretreated with NLGN3 (5 or 25 ng/mL) or vehicle control (PBS, “Veh”) for 30 min, maintained under oxygen glucose deprivation (OGD) for 4 h and then re-oxygenation (OGD/R) for 48 h, cell viability and death were tested by CCK-8 (**B**, **F**) and Trypan blue staining (**C**, **G**) assays, respectively, expression of listed proteins was shown (**D**, **H**). “Mock” stands for the mock treatment (norm-oxygenated medium with glucose). Blotting data was the representative of five replicate experiments. Data were presented as mean ± standard deviation (SD, *n* = 5). *P < 0.05 vs. “Mock” cells. ^#^*P* < 0.05 vs OGD/R with “Veh” pretreatment. Each experiment was repeated five times and similar results were obtained.

NLGN3 was shown to activate Akt-mTOR, Erk-MAPK, and other signaling cascades in glioma cells [18, 19, 36, 50]. We first tested whether NLGN3 could activate the signalings in neuronal cells. The differentiated SH-SY5Y neuronal cells were treated with NLGN3 at gradually increased concentrations, from 1-100 ng/mL. Western blotting assays were employed to examine the signaling proteins. Results showed that NLGN3, in a concentration-dependent manner, increased phosphorylation of Akt (Ser-473), p70S6 kinase 1 (S6K1), and Erk1/2 in SH-SY5Y cells (Fig. 1A). In SH-SY5Y cells, NLGN3-induced Akt-mTOR and Erk activation was significant at 5–100 ng/mL, but was invalid at 1 ng/mL (Fig. 1A).

To support the potential neuron protective activity of NLGN3, SH-SY5Y neuronal cells were first pretreated (for 30 min) with NLGN3 (5 or 25 ng/mL), followed by OGD/R procedure [10]. In detail, the differentiated SH-SY5Y cells were subject to oxygen-glucose deprivation (OGD) procedure for 4 h and then cultivated under the complete medium (“re-oxygenation”, OGD/R). There was a time-dependent response following OGD/R stimulation in SH-SY5Y cells. Viability (CCK-8 OD) reduction and cell death started 24 h after OGD/R stimulation and were most significant at 48 h (Fig. S1A, B). Importantly, NLGN3 pretreatment largely ameliorated OGD/R-induced neuronal cell viability reduction (Fig. 1B) and cell death (Fig. 1C). Single treatment with NLGN3, at tested concentrations (5 or 25 ng/mL for 48 h), did not significantly alter cell viability and Trypan blue staining in SH-SY5Y cells (Fig. 1B, C). Expression of PSD-95, the neuronal marker, was also downregulated in OGD/R-treated SH-SY5Y cells (Fig. 1D), which was partially restored by pretreatment with NLGN3 (Fig. 1D).

The potential effect of NLGN3 in the primary murine cortical neurons was studied next. Western blotting assay results, Fig. 1E, demonstrated that NLGN3 (25 ng/mL, 15′) treatment potently increased phosphorylation of Akt (Ser-473), S6K1 and Erk1/2 in murine neurons. OGD/R stimulation time-dependently decreased viability in cortical neurons and was most significant at 48 h (Figure S1C). Significantly, OGD/R-induced viability reduction (Fig. 1F) and cell death (Fig. 1G) were largely attenuated by NLGN3 (25 ng/mL) pretreatment. NLGN3 treatment alone was ineffective (Fig. 1F, G). Downregulation of PSD-95 by OGD/R was also largely ameliorated by NLGN3 in cortical neurons (Fig. 1H). Thus, NLGN3 activated Akt-mTOR and Erk signalings and ameliorated OGD/R-induced neuronal cell death.

### NLGN3 ameliorates OGD/R-induced neuronal cell apoptosis

**Fig. 2:**
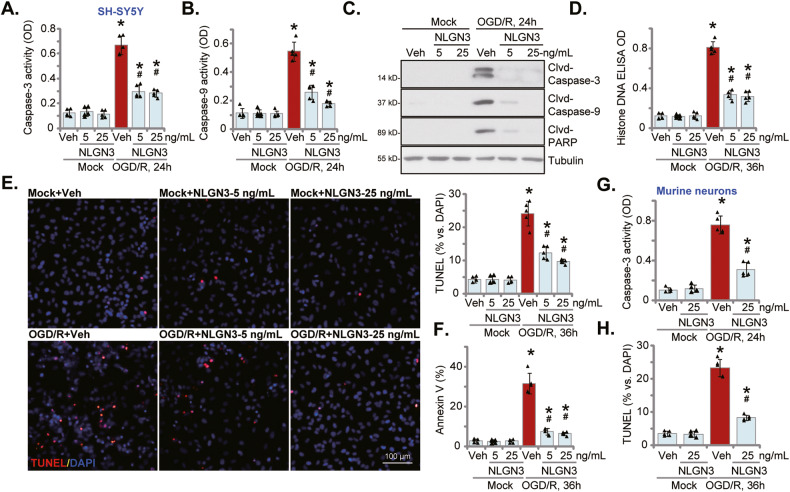
NLGN3 ameliorates OGD/R-induced neuronal cell apoptosis. The differentiated SH-SY5Y neuronal cells (**A**–**F**) or the primary murine cortical neurons (**G**, **H**) were pretreated with NLGN3 (5 or 25 ng/mL) or vehicle control (PBS, “Veh”) for 30 min, maintained under oxygen glucose deprivation (OGD) for 4 h and then re-oxygenation (“OGD/R”) for the applied time periods; Caspase-PARP activation (**A**–**C** and **G**) and Histone-bound DNA contents (ELISA assays, **D**) were tested; Cell apoptosis was examined by nuclear TUNEL staining (**E**, **H**) and Annexin V-PI FACS (**F**) assays, with results quantified. “Mock” stands for the mock treatment (norm-oxygenated medium with glucose). Blotting data was the representative of five replicate experiments. Data were presented as mean ± standard deviation (SD, *n* = 5). *P < 0.05 vs. “Mock” cells. ^#^*P* < 0.05 vs. OGD/R with “Veh” pretreatment. Each experiment was repeated five times and similar results were obtained. Scale Bar = 100 μm.

OGD/R could provoke apoptosis in neuronal cells [41, 42, 51, 52]. We next examined whether NLGN3 could affect cell apoptosis. In the differentiated SH-SY5Y neuronal cells, OGD/R stimulation-induced Caspase-3 activation was significant at 12 h after OGD/R and was most significant at 24 h (Figs. S1D and 2A). OGD/R also robustly increased the caspase-9 activity (Fig. 2B). In addition, cleavages of caspase-3, caspase-9 and poly (ADP ribose) polymerase 1 (PARP) were detected in OGD/R-stimulated SH-SY5Y cells (Fig. 2C). Moreover, OGD/R increased Histone-bound DNA breaks (ELISA assays, Fig. 2D) in SH-SY5Y cells. Importantly, pretreatment with NLGN3 (5 or 25 ng/mL, 30 min pretreatment) largely ameliorated OGD/R-induced caspase-PARP activation (Fig. 2A–C) and Histone-bound DNA accumulation (Fig. 2D) in SH-SY5Y cells.

In SH-SY5Y cells, OGD/R stimulation induced significant apoptosis activation, which was evidenced by significantly increased TUNEL-positive nuclei ratio (Figs. S1E and 2E) and Annexin V-positive staining percentage (Fig. 2F). Such pro-apoptotic actions by OGD/R were largely attenuated by NLGN3 pretreatment (Fig. 2E, F). NLGN3 single treatment, as expected, failed to induce caspase-apoptosis activation in SH-SY5Y cells (Fig. 2A–F). In the primary murine cortical neurons, NLGN3 pretreatment (25 ng/mL, 30 min pretreatment) largely inhibited OGD/R-induced caspase-3 activation (Fig. 2G) and apoptosis activation (Fig. 2H), the latter was tested by the increased nuclear TUNEL ratio (Fig. 2H). These results together showed that NLGN3 ameliorated OGD/R-induced neuronal cell apoptosis.

### NLGN3 ameliorates OGD/R-induced oxidative injury and programmed necrosis in neuronal cells

**Fig. 3:**
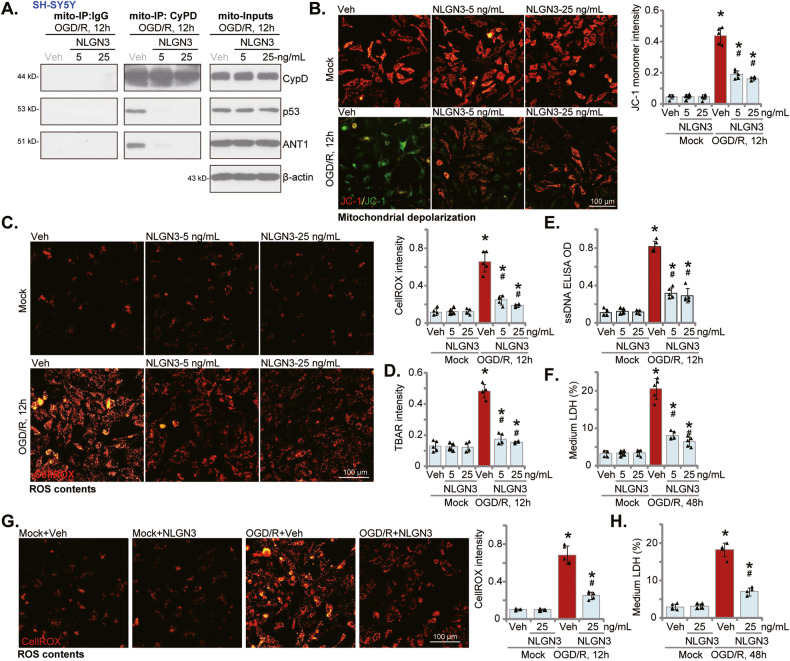
NLGN3 ameliorates OGD/R-induced oxidative injury and programmed necrosis in neuronal cells. The differentiated SH-SY5Y neuronal cells (**A**–**F**) or the primary murine cortical neurons (**G**, **H**) were pretreated with NLGN3 (5 or 25 ng/mL) or vehicle control (PBS, “Veh”) for 30 min, maintained under oxygen glucose deprivation (OGD) for 4 h and then re-oxygenation (“OGD/R”) for the applied time periods, the association and expression of CypD-p53-ANT1 complex in the mitochondrial lysates were shown (**A**); Mitochondrial depolarization and ROS production were tested by JC-1 staining (**B**) and CellROX staining (**C**, **G**) assays, respectively; Lipid peroxidation was examined by the TBAR activity assays (**D**), with single strand DNA (ssDNA) contents measured by ELISA assays (**E**); Cell necrosis was tested by measuring medium LDH contents (**F**, **G**). “Mock” stands for the mock treatment (norm-oxygenated medium with glucose). Blotting data was the representative of five replicate experiments. Data were presented as mean ± standard deviation (SD, *n* = 5). *P < 0.05 vs. “Mock” cells. ^#^*P* < 0.05 vs. OGD/R with “Veh” pretreatment. Each experiment was repeated five times and similar results were obtained. Scale Bar = 100 μm.

Besides apoptosis, studies have also shown that OGD/R could provoke programmed necrosis cascade in neuronal cells [41, 52] and other cells [53–55]. In line with these findings, we demonstrated that OGD/R induced programmed necrosis activation in SH-SY5Y neuronal cells, causing mitochondrial CypD-p53-ANT1 association (Fig. 3A), mitochondrial depolarization (JC-1 green monomers accumulation, Fig. 3B) and ROS production (the CellROX intensity increasing, Fig. 3C). Significantly, such actions by OGD/R were largely inhibited by NLGN3 pretreatment (Fig. 3A–C). Note that ROS production, or CellROX intensity increasing, was significant 6 h after OGD/R stimulation and was most significant at 12 h (Fig. S1F). In addition, NLGN3 significantly attenuated OGD/R-induced lipid peroxidation (reflected by the TBAR activity increase, Fig. 3D), ssDNA accumulation (indicating DNA breaks, Fig. 3E).

OGD/R stimulation time-dependently induced cell necrosis in SH-SY5Y cells and medium LDH release started at 24 h after OGD/R and was most significant at 48 h (Fig. S1G). NLGN3 pretreatment remarkably attenuated OGD/R-induced medium LDH release IN SH-SY5Y cells (Fig. 3F). The single NLGN3 treatment, unsurprisingly, did not provoke programmed necrosis cascade in SH-SY5Y cells (Fig. 3A–F). In the primary murine cortical neurons, NLGN3 pretreatment (25 ng/mL, 30 min pretreatment) largely inhibited OGD/R-induced ROS production (CellROX intensity increase, Fig. 3G). Moreover, OGD/R-induced medium LDH release, indicating cell necrosis, was also attenuated by NLGN3 pretreatment in primary neurons (Fig. 3H). These results showed that NLGN3 inhibited OGD/R-induced oxidative injury and programmed necrosis in SH-SY5Y cells, further supporting its neuroprotective activity.

### NLGN3-induced neuronal protection against OGD/R requires Akt activation

**Fig. 4:**
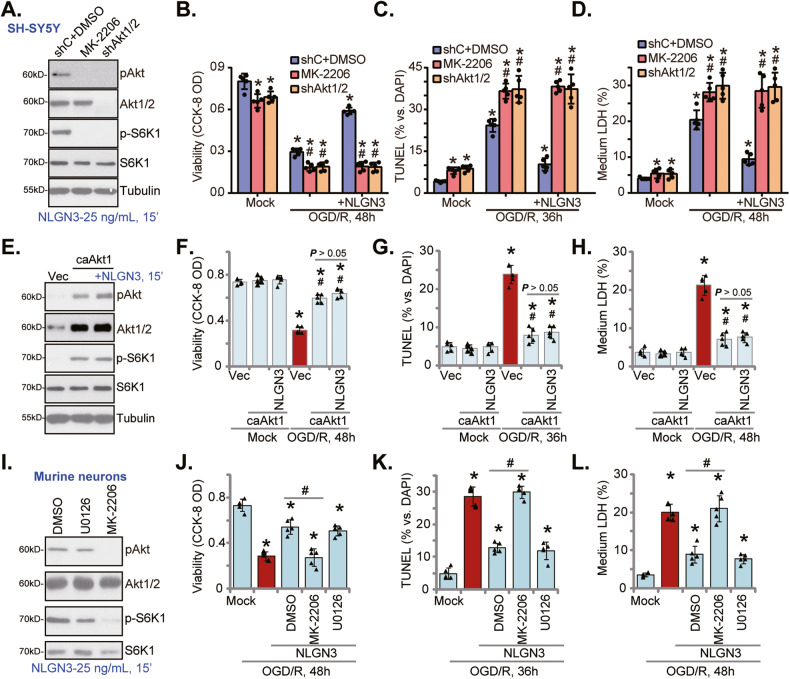
NLGN3-induced neuronal protection against OGD/R requires Akt activation. SH-SY5Y neuronal cells were pretreated with MK-2206 (10 μM, for 30 min) or stably transduced with the Akt1/2 shRNA (“shAkt1/2”), followed by NLGN3 (25 ng/mL, 15 min) treatment, expression of listed proteins was shown (**A**). Alternatively, cells were maintained under oxygen glucose deprivation (OGD) for 4 h and then re-oxygenation (“OGD/R”) for the applied time periods, cell viability, apoptosis and necrosis were tested by CCK-8 (**B**), nuclear TUNEL staining (**C**), and medium LDH release (**D**) assays, respectively. SH-SY5Y cells, stably expressing the constitutively-active Akt1 (ca-Akt1, S473D) or the empty vector (“Vec”), were treated with or without NLGN3 (25 ng/mL, 15 min), expression of listed proteins was shown (**E**). Alternatively, cells were subjected to the OGD/R stimulation, cell viability (**F**), apoptosis (**G**), and necrosis (**H**) were tested similarly. The primary murine cortical neurons were pretreated with U0126 (10 μM, for 30 min), MK-2206 (10 μM, for 30 min) or the vehicle control (0.1% DMSO), followed by NLGN3 (25 ng/mL, 15 min) treatment, expression of listed proteins was shown (**I**). Neurons were maintained under oxygen glucose deprivation (OGD) for 4 h and then re-oxygenation (“OGD/R”) for the applied time periods, cell viability (**J**), apoptosis (**K**), and necrosis (**L**) were tested similarly. “shC+DMSO” stands for cells with scramble control shRNA plus 0.1% DMSO treatment (**A**–**D**). “Mock” stands for the mock treatment (norm-oxygenated medium with glucose). Blotting data was the representative of five replicate experiments. Data were presented as mean ± standard deviation (SD, *n* = 5). *P < 0.05 vs. “Mock” cells. ^#^*P* < 0.05 vs. OGD/R treatment in control cells. ^#^*P* < 0.05 (**J**–**L**). Each experiment was repeated five times and similar results were obtained.

Since Akt is a pivotal pro-survival signaling cascade in neurons [56–60], we therefore tested whether activation of Akt was required for NLGN3-mediated neuroprotection against OGD/R. MK-2206, an Akt-specific inhibitor [61–63], was utilized to block Akt activation. Alternatively, the Akt1/2 shRNA lentiviral particles were transfected to SH-SY5Y neuronal cells. Stable cells, “shAkt1/2”, were established after puromycin selection, showing depleted Akt1/2 (Fig. 4A). As shown, NLGN3 (25 ng/mL, 15 min)-induced phosphorylation of Akt and S6K1 was almost blocked by MK-2206 and shAkt1/2 in SH-SY5Y cells (Fig. 4A). Functional studies demonstrated that MK-2206 or shAkt1/2 exacerbated OGD/R-induced viability (CCK-8 OD) reduction (Fig. 4B), cell apoptosis (tested by the TUNEL-positive nuclei ratio increase, Fig. 4C) and necrosis (medium LDH release, Fig. 4D). Importantly, NLGN3-induced neuronal protection against OGD/R was completely abolished by MK-2206 or shAkt1/2 in SH-SY5Y cells (Fig. 4B–D). Specifically, after Akt inhibition or silencing OGD/R-induced cell viability reduction (Fig. 4B), apoptosis (Fig. 4C), and necrosis (Fig. 4D) were not alleviated by NLGN3. These results supported that NLGN3-induced neuronal protection against OGD/R required Akt activation.

To further support our hypothesis, a constitutively-active Akt1 (“caAkt1”, S473D) construct (from Dr. Li at Wenzhou Medical University [64]) was stably transduced to SH-SY5Y cells (Fig. 4E), which resulted in sustained Akt-S6K1 activation (Fig. 4E). As shown, caAkt1 largely inhibited OGD/R-induced viability reduction (Fig. 4F), apoptosis (Fig. 4G) and necrosis (Fig. 4H) in SH-SY5Y cells. Intriguingly, adding NLGN3 failed to further increase Akt-S6K1 phosphorylation in caAkt1-expressing SH-SY5Y cells (Fig. 4E). Neither did it offer additional neuronal protection against OGD/R (Fig. 4F–H). Therefore, NLGN3 was invalid against OGD/R-induced cytotoxicity in SH-SY5Y cells with caAkt1 (Fig. 4F–H).

In the primary murine cortical neurons, MK-2206, the Akt-specific inhibitor, blocked NLGN3-induced Akt-S6K1 activation (Fig. 4I). In the presence of MK-2206, OGD/R-induced viability reduction (Fig. 4J), apoptosis (Fig. 4K) and necrosis (Fig. 4L) were not attenuated by NLGN3. Conversely, U0126, the Erk inhibitor, failed to inhibit NLGN3-induced neuronal protection against OGD/R (Fig. 4J–L). These results further supported that Akt activation is essential for NLGN3-induced neuronal protection against OGD/R.

### Gαi1 and Gαi3 mediate NLGN3-induced Akt signaling activation in neuronal cells

**Fig. 5:**
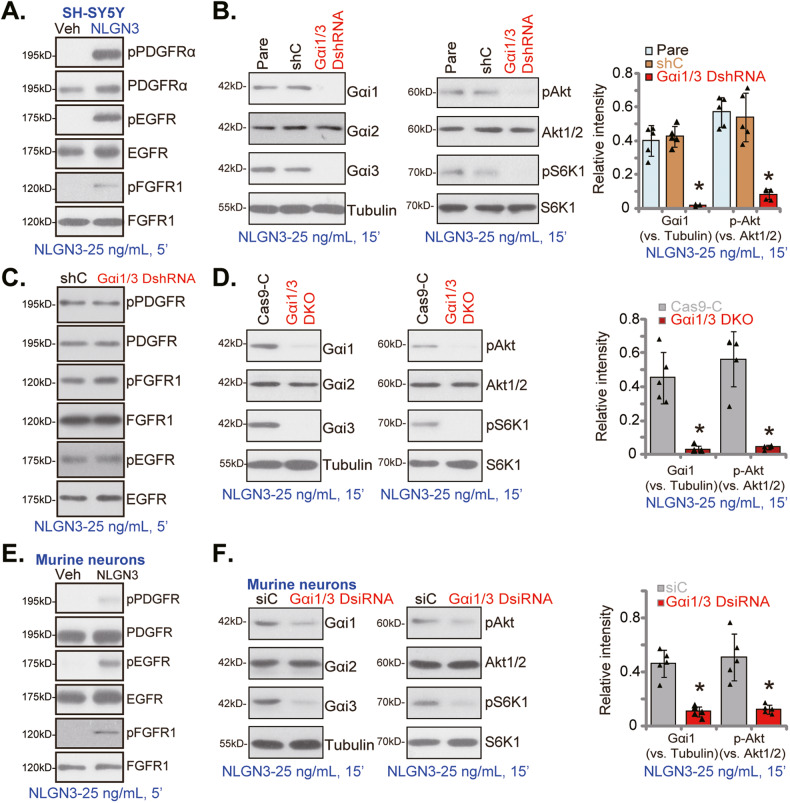
Gαi1 and Gαi3 mediate NLGN3-induced Akt signaling activation in neuronal cells. SH-SY5Y neuronal cells (**A**) or the primary murine cortical neurons (**E**) were treated with NLGN3 (25 ng/mL) for 5 min, expression of listed proteins was tested by Western blotting assays. The stable SH-SY5Y neuronal cells, with the Gαi1 shRNA plus the Gαi3 shRNA (“Gαi1/3 DshRNA”) or the scramble control shRNA (“shC”), were treated with NLGN3 (25 ng/mL) for applied time periods, expression of the listed proteins in total cell lysates was tested (**B**, **C**). The stable SH-SY5Y cells, with the Gαi1 plus Gαi3 CRISPR/Cas9 KO constructs (“Gαi1/3 DKO”) or the CRISPR/Cas9 control construct (“Cas9-C”), were treated with NLGN3 and tested by Western blotting of listed proteins (**D**). The primary murine cortical neurons were transfected with the Gαi1 siRNA plus the Gαi3 siRNA (“Gαi1/3 DsiRNA”) or the scramble control siRNA (“siC”), treated with NLGN3 (25 ng/mL), and tested by western blotting of listed proteins (**F**). Blotting data was the representative of five replicate experiments. “Pare” stands for the parental control cells. Data were presented as mean ± standard deviation (SD, *n* = 5). *P < 0.05 vs. the corresponding control cells. Each experiment was repeated five times and similar results were obtained.

Studies have shown that NLGN3-induced phosphorylation of several key RTKs in glioma cells [18–20]. We have previously demonstrated that Gαi1/3 associated with ligand-activated RTKs to mediate downstream signal transduction [30, 32–34]. A very recent study of our group has also shown Gαi1/3 are key proteins mediating NLGN3-induced signaling in glioma cells [36]. Extending these studies, we found that NGLN3 can induce phosphorylation of multiple RTKs (EGFR, FGFR1, and PDGFR) in SH-SY5Y neuronal cells (Fig. 5A).

To silence Gαi1 and Gαi3, SH-SY5Y neuronal cells were infected with the Gαi1 shRNA-expressing lentivirus and the Gαi3 shRNA-expressing lentivirus (see our previous studies [34, 36, 38]), stable cells were established after selection by puromycin: “Gαi1/3 DshRNA” cells. As shown, Gαi1 and Gαi3 protein expression was robustly downregulated in Gαi1/3 DshRNA SH-SY5Y cells (Fig. 5B), leaving Gαi2 protein expression unaffected (Fig. 5B). NLGN3 (25 ng/mL, 15 min)-induced phosphorylation of Akt and S6K1 was largely inhibited by Gαi1/3 DshRNA (Fig. 5B). The scramble control shRNA (“shC”), unsurprisingly, did not alter Gαi1/3 expression and NLGN3-induced signaling in SH-SY5Y cells (Fig. 5B). We have previously shown that Gαi1/3 association is essential for RTKs endocytosis and downstream signal activation [33, 34]. Expression of RTKs and NLGN3-induced phosphorylation of RTKs were not affected by Gαi1/3 double shRNA (Fig. 5C). These results implied that Gαi1/3 are required for NLGN3-induced RTKs downstream Akt signaling transduction.

To further support the requirement of Gαi1/3 in NLGN3-induced Akt signaling in neuronal cells, the CRISPR/Cas9 strategy was applied. The Cas9-expressing SH-SY5Y cells were further transfected with the CRISPR/Cas9-Gαi1-KO construct plus the CRISPR/Cas9-Gαi3-KO construct (see the previous studies [34, 36, 38]). Single stable SH-SY5Y cells with Gαi1/3 double KO (“Gαi1/3 DKO”) were established after puromycin selection and Gαi1/3 KO screening. As shown protein expression of Gαi1 and Gαi3, but not Gαi2, was depleted in Gαi1/3 DKO SH-SY5Y cells (Fig. 5D), where NLGN3 (25 ng/mL, 15 min)-induced phosphorylation of Akt and S6K1 was almost completely blocked (Fig. 5D).

In the primary murine cortical neurons, NLGN3 treatment (25 ng/mL, 5 min) similarly induced phosphorylation of multiple RTKs (PDGFRα, EGFR, and FGFR1) (Fig. 5E). The siRNA strategy was employed to silence Gαi1 and Gαi3. The Gαi1 siRNA and the Gαi3 siRNA (“Gαi1/3-DsiRNA”) were co-transfected to primary murine neurons for 48 h, resulting in significant Gαi1 and Gαi3 downregulation (Fig. 5F). Consequently, Gαi1/3-DsiRNA largely inhibited NLGN3-induced Akt-S6K1 activation in primary murine neurons (Fig. 5F).

### NLGN3-induced neuronal protection against OGD/R requires Gαi1/3

**Fig. 6:**
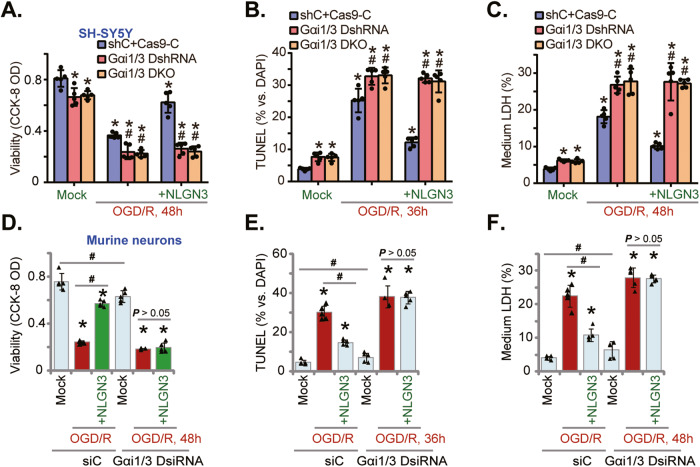
NLGN3-induced neuronal protection against OGD/R requires Gαi1/3. SH-SY5Y neuronal cells, with the Gαi1 plus Gαi3 shRNA (“Gαi1/3 DshRNA”), the Gαi1 plus Gαi3 CRISPR/Cas9 KO constructs (“Gαi1/3 DKO”), or the scramble control shRNA plus the CRISPR/Cas9 control construct (“shC+Cas9-C”), were pretreated with NLGN3 (25 ng/mL, for 30 min), cells were then maintained under oxygen glucose deprivation (OGD) for 4 h and then re-oxygenation (“OGD/R”) for the applied time periods; Cell viability, apoptosis and necrosis were tested by CCK-8 (**A**), nuclear TUNEL staining (**B**), and medium LDH release (**C**) assays, respectively. The primary murine cortical neurons, transfected with the Gαi1 siRNA plus the Gαi3 siRNA (“Gαi1/3 DsiRNA”) or the scramble control siRNA (“siC”), were pretreated with NLGN3 (25 ng/mL, for 30 min), cells were maintained under oxygen glucose deprivation (OGD) for 4 h and then re-oxygenation (“OGD/R”) for the applied time periods, cell viability (**D**), apoptosis (**E**), and necrosis (**F**) were tested similarly. “Mock” stands for the mock treatment (norm-oxygenated medium with glucose). Data were presented as mean ± standard deviation (SD, *n* = 5). *P < 0.05 vs. “Mock” cells. ^#^*P* < 0.05 vs. OGD/R treatment in the corresponding control cells (**A**-**C**). ^#^*P* < 0.05 (**D**-**F**). Each experiment was repeated five times and similar results were obtained.

Since Gαi1/3 are essential for NLGN3-induced Akt-S6K1 activation in SH-SY5Y cells and primary murine neurons, we therefore hypothesized that Gαi1/3 should be required for NLGN3-mediated neuroprotection against OGD/R. As shown, in the Gαi1/3 DshRNA SH-SY5Y cells and the Gαi1/3 DKO SH-SY5Y cells (Fig. 5), OGD/R-induced viability (CCK-8 OD) reduction (Fig. 6A), apoptosis activation (the TUNEL-positive nuclei ratio increase, Fig. 6B) and necrosis (by measuring medium LDH release, Fig. 6C) were augmented (*P* < 0.05 vs. control cells). More importantly, in Gαi1/3-silenced or Gαi1/3-DKO SH-SY5Y cells, pretreatment with NLGN3 (25 ng/mL) failed to inhibit OGD/R-induced cell death (Fig. 6A–C). In the primary murine cortical neurons OGD/R-induced viability (CCK-8 OD) reduction (Fig. 6D), apoptosis activation (TUNEL assays, Fig. 6E) and necrosis (LDH assays, Fig. 6F) were augmented after Gαi1/3 DsiRNA. With Gαi1/3 silencing, NLGN3-induced neuronal protection against OGD/R was abolished (Fig. 6D–F). These results clearly showed that NLGN3-induced neuronal protection against OGD/R requires Gαi1/3.

### ADAM10 inhibition blocks NLGN3 cleavage, exacerbating MCAO-induced ischemic brain injury in mice

**Fig. 7:**
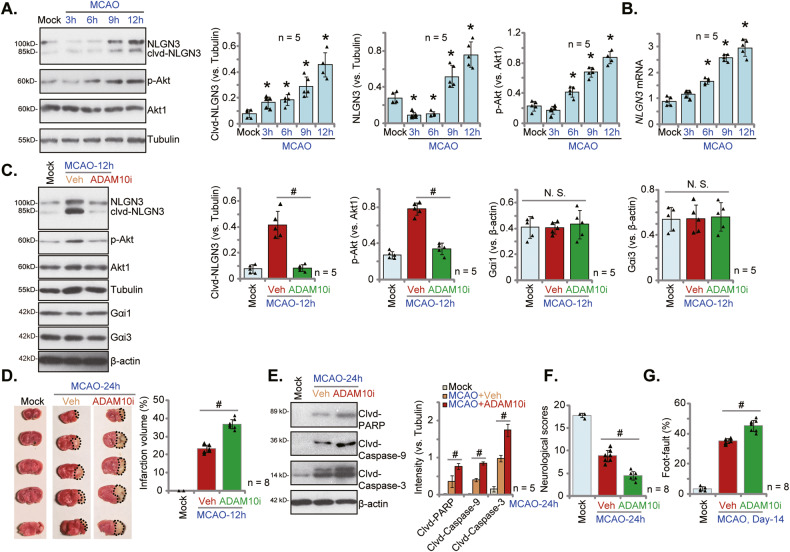
*ADAM10* inhibition blocks NLGN3 cleavage, exacerbating MCAO-induced ischemic brain injury in mice. C57BL/6J mice were subject to MCAO procedure for applied time periods, and the ischemic penumbra brain regions were isolated. Expression of listed genes and proteins in the brain tissues were tested (**A**, **B**). GI254023X, the ADAM10 inhibitor (“ADAM10i”, 100 μM, at a rate of 1 μL/min for 5 min), or the vehicle control (“Veh”) was injected to the lateral ventricle of the C57BL/6 J mice. After 6 h mice were subject to the MCAO procedure, the ischemic penumbra brain regions were isolated 12 h after MCAO and expression of listed proteins in the brain tissues was tested (**C**). TTC staining was employed to stain the ischemic region 24 h after MCAO and results were quantified (**D**). Expression of the apoptosis-associated proteins was tested as well (**E**).Other mice were subject to the behavior tests, the neurological scores were recorded (**F**, at 24 h) and foot-fault tests (**G**, at Day-14) were performed. Data were presented as mean ± standard deviation (SD). *P < 0.05 vs. “Mock” (**A**, **B**). ^#^*P* < 0.05. ^“^N.S.” stands for non-statistical difference (*P* > 0.05). In each group there were five to eight mice (*n* = 5/8).

Next, we studied the potential neuroprotective effect of NLGN3 in vivo. We first examined whether NLGN3 cleavage and expression were altered in the brain tissues with cerebral ischemia/re-perfusion. MCAO was performed in the mice. The ischemic penumbra brain tissues were then collected 3 h, 6 h, 9 h, and 12 h after MCAO. As shown, cleaved-NLGN3 levels were significantly increased in the ischemic penumbra brain tissues of MCAO mice (Fig. 7A). Moreover, phosphorylated-Akt level was also increased, indicating Akt activation (Fig. 7A). NLGN3 cleavage and Akt activation in the brain tissues of MCAO mice were time-dependent (Fig. 7A). Remarkably, *NLGN3* mRNA and non-cleaved NLGN3 protein levels in the brain tissues were also significantly increased 9 h and 12 h after MCAO (Fig. 7A). These results implied that MCAO-induced NLGN3 cleavage and secretion, and increased its expression in mouse brain tissues.

NLGN3 is mainly cleaved by ADAM10 (A Disintegrin and Metalloproteinase 10) in neurons [19]. ADAM10 inhibition was shown to prevent cleavage and secretion of *NLGN3* into the microenvironment [19]. Thus, an ADAM10 inhibitor GI254023X (ADAM10i) was injected to the lateral ventricle. After 6 h, mice were then subject to the MCAO procedure. As demonstrated, MCAO-induced NLGN3 cleavage and Akt activation in the ischemic penumbra brain tissues were completely blocked by ADAM10i (Fig. 7C). Gαi1 and Gαi3 protein expression was unchanged by MCAO or with ADAM10i administration (Fig. 7C). TTC staining assay results showed that MCAO-induced ischemic brain injury was intensified following the lateral ventricle injection of ADAM10i (Fig. 7D), as the infarct area was significantly enlarged (Fig. 7D). Moreover, ADAM10i intensified MCAO-induced apoptosis, and cleavages of caspase-3, caspase-9 and PARP in the ischemic penumbra brain tissues were increased after ADAM10 injection (Fig. 7E). In addition, as compared to vehicle control MCAO mice, the neurological scores were worse in ADAM10i-injected MCAO mice (Fig. 7F). The foot-fault tests were carried out 14 days after MCAO. Results showed that MCAO-induced foot-fault was significantly increased in ADAM10i-injected mice (Fig. 7G). These results together implied that *ADAM10* inhibition blocked MCAO-induced NLGN3 cleavage and secretion, exacerbating ischemic brain injury in mice.

### Neuronal silencing of NLGN3-Gαi1/3 exacerbates MCAO-induced ischemic brain injury in mice

**Fig. 8:**
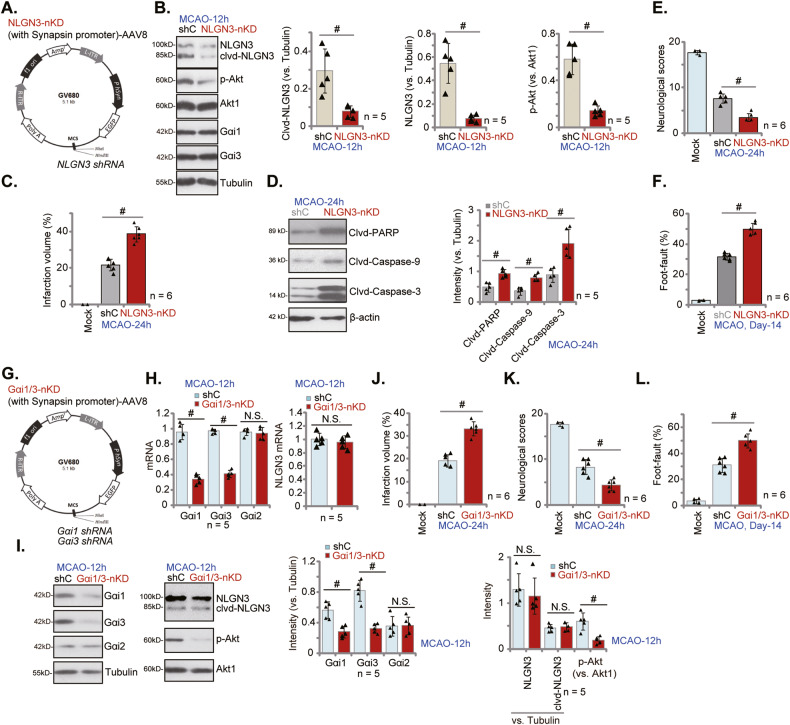
Neuronal silencing of NLGN3-Gαi1/3 exacerbates MCAO-induced ischemic brain injury in mice. NLGN3 shRNA-expressing AAV (NLGN3 shRNA-hSyn-AAV8, “NLGN3-nKD”, **A**), the Gαi1 shRNA-expressing adenovirus plus the Gαi3 shRNA-expressing adenovirus (hSyn-AAV8, “Gαi1/3-nKD”, **G**) or the scramble control shRNA-expressing adenovirus (hSyn-AAV8, “shC”) were injected to the lateral ventricle of the mice; After 20 days, the mice were subject to MCAO procedure. After indicated time periods, the ischemic penumbra brain regions were isolated and expression of listed mRNAs and proteins were tested (**B**, **D**, **H**, **I**). TTC staining was employed to stain the ischemic region and results were quantified (**C**, **J**). Other mice were subject to the behavior tests, the neurological scores were recorded (**E**, **K**, at 24 h) and foot-fault tests (**F**, **L**, at Day-14) were performed. Data were presented as mean ± standard deviation (SD). ^#^*P* < 0.05. “N.S.” stands for non-statistical difference (*P* > 0.05). In each group there were five to eight mice (*n* = 5/8).

To further support the neuroprotective effect of NLGN3 in vivo, the NLGN3 shRNA-expressing adenovirus (AAV8 construct, containing the Synapsin promoter region) (Fig. 8A) was injected to the lateral ventricle, aiming to conditional knockdown of neuronal NLGN3: NLGN3-nKD. The control mice group received lateral ventricle injection of scramble control shRNA adenovirus (“shC”, AAV8 construct, containing the Synapsin promoter region) (Fig. 8A). After 20 days, the NLGN3-nKD mice and the shC group mice were subject to the same MCAO procedure. Twelve hour after, the ischemic penumbra brain tissues were separated and tested. As demonstrated, MCAO-induced NLGN3 cleavage and expression, as well as Akt activation, were largely inhibited in the NLGN3-nKD group mice (Fig. 8B). Gαi1 and Gαi3 protein expression were unchanged by NLGN3 shRNA (Fig. 8B). NLGN3-nKD exacerbated MCAO-induced ischemic brain injury, and the infarct area was significantly enlarged (Fig. 8C). Moreover, neuronal silencing of NLGN3 enhanced MCAO-induced apoptosis. Levels of cleaved-caspase-3, cleaved-caspase-9, and cleaved-PARP were increased in the ischemic penumbra brain tissues of NLGN3-nKD mice (Fig. 8D). The neurological scores were much worse in MCAO NLGN3-nKD mice as compared to shC group mice (Fig. 8E). In addition, 14 days after the MCAO procedure, the foot-fault ratio was significantly higher after neuronal silencing of NLGN3 (Fig. 8F). These results clearly supported that neuronal silencing of NLGN3 exacerbated MCAO-induced ischemic brain injury.

The in vitro experimental results have shown that Gαi1 and Gαi3 mediated NLGN3-induced Akt activation and neuroprotection against OGD/R. We therefore proposed that neuronal silencing of Gαi1 and Gαi3 should also exacerbate MCAO-induced ischemic brain injury in mice. Therefore, the Gαi1 shRNA-expressing adenovirus and the Gαi3 shRNA-expressing adenovirus (AAV8 construct, containing the Synapsin promoter region) were co-injected to the lateral ventricle for 20 days, leading to neuronal conditional knockdown of Gαi1 and Gαi3 (“Gαi1/3-nKD”) (Fig. 8G). Control shC group mice and Gαi1/3-nKD mice were subject to the same MCAO procedure. After 12 h, the ischemic penumbra brain tissues were isolated. qRT-PCR and Western blotting assay results confirmed *Gαi1/3* mRNA and protein downregulation in Gαi1/3-nKD mice (Fig. 8H and I). Expression of Gαi2 and NLGN3 (total and cleaved) was however unchanged (Fig. 8H, I). Neuronal silencing of Gαi1 and Gαi3 largely inhibited Akt phosphorylation in brain tissues of MCAO mice (Fig. 8I). The quantified TTC staining results found that neuronal silencing of Gαi1 and Gαi3 augmented MCAO-induced ischemic brain injury and enlarged the infarct area (Fig. 8J). The neurological scores, recorded 24 h after MCAO, were worse in Gαi1/3 nKD mice as compared to the shC group mice (Fig. 8K). In addition, MCAO-induced foot-fault was remarkably increased with neuronal silencing of Gαi1/3 (Fig. 8L). Therefore, neuronal silencing of Gαi1 and Gαi3 intensified MCAO-induced ischemic brain injury in mice.

### Neuronal overexpression of NLGN3 alleviates MCAO-induced ischemic brain injury in mice

**Fig. 9:**
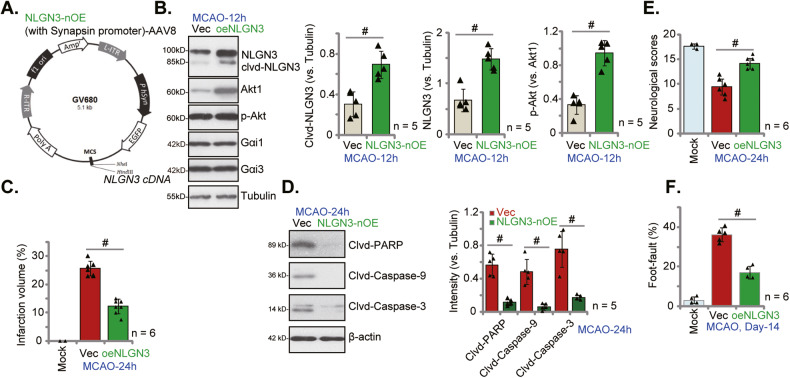
Neuronal overexpression of NLGN3 alleviates MCAO-induced ischemic brain injury in mice. The NLGN3-overexpressing adenovirus containing the Synapsin promoter region (*NLGN3* cDNA-hSyn-AAV8) was injected to the lateral ventricle of the mice (“NLGN3-nOE” group) (**A**); The control group mice received the hSyn-AAV8 empty vector adenovirus (“Vec”) injection; After 20 days, the mice were subject to the same MCAO procedure. After indicated time periods, the ischemic penumbra brain regions were isolated and expression of listed proteins in the brain tissues were tested (**B**, **D**). TTC staining was employed to stain the ischemic region and results were quantified (**C**). Other mice were subject to the behavior tests, the neurological scores were recorded (**E**, at 24 h) and foot-fault tests (**F**, at Day-14) were performed. Data were presented as mean ± standard deviation (SD). ^#^*P* < 0.05. In each group there were five to eight mice (*n* = 5/6).

We further hypothesized that neuronal overexpression of NLGN3 could possibly inhibit ischemic brain injury in mice. Therefore, the NLGN3-overexpressing adenovirus (AAV8, containing the Synapsin promoter region, Fig. 9A) was injected to the lateral ventricle, causing neuronal NLGN3 overexpression (“NLGN3-nOE”) after 20 days. Control mice were injected with hSyn-AAV8-empty vector adenovirus (“Vec”). Mice were subject the same MCAO procedure. The ischemic penumbra brain tissues were then isolated and tested. As shown *NLGN3* expression and cleavage were both significantly increased in ischemic penumbra brain regions in the NLGN3-nOE mice (Fig. 9B). Akt activation was increased as well (Fig. 9B). Neuronal overexpression of NLGN3 significantly inhibited MCAO-induced ischemic brain injury in mice. The infarct area was decreased in MCAO NLGN3-nOE mice (Fig. 9C) and cleavages of apoptosis proteins were remarkably decreased (Fig. 9D). Remarkably, in MCAO NLGN3-nOE mice, the neurological scores were significantly improved as compared to Vec control mice (Fig. 9E), and the foot-fault ratio was remarkably decreased (Fig. 9F). These results clearly showed that NLGN3-nOE protected against MCAO-induced ischemic brain injury in mice.

## Discussion

NLGN3 was shown to induce expression of synaptic genes required for neuron-glioma synapse formation [65, 66]. It was identified as the primary factor responsible for neuronal-driven glioma progression [18–21]. NLGN3 can activate multiple RTKs and the downstream cascades (i.e. PI3K-Akt-mTOR), promoting cell survival and growth in glioma cells [18–20]. The current study showed that MCAO procedure induced NLGN3 cleavage and secretion, and significantly increased its expression in mouse brain tissues. NLGN3 exerted significant neuroprotective activity and protected neuronal cells from ischemia-reperfusion injury.

Besides apoptosis, recent studies have shown that OGD/R could induce programmed necrosis in neuronal cells and other cells. Tang et al., showed that Ginseng Rh2 protected endometrial cells from OGD/R-induced oxidative injury via blocking CypD-dependent programmed necrosis pathway [55]. Xu et al., reported that microRNA-1203 silenced CypD to protect endometrial cells from OGD/R-induced programmed necrosis [67]. In myocardial cells, a novel Akt activator SC79 largely inhibited OGD/R-induced mitochondrial depolarization, CypD-p53-ANT1 association, oxidative injury, and programmed necrosis [68]. Liu et al., showed that K6PC-5, a novel sphingosine kinase 1 (SphK1) activator, activated SphK1-Nrf2 cascade and inhibited OGD/R-induced programmed necrosis in SH-SY5Y neuronal cells [41].

We showed that NLGN3, aside from apoptosis inhibition, simultaneously suppressed OGD/R-induced programmed necrosis, thereby exerting significant neuroprotective activity. In SH-SY5Y cells and the primary murine cortical neurons, OGD/R-induced mitochondrial CypD-p53-ANT1 association, mitochondrial depolarization, ROS production, oxidative injury and cell necrosis were largely ameliorated by NLGN3 pretreatment. NLGN3-induced anti-programmed necrosis mechanism could be one primary reason of its superior neuroprotective activity against OGD/R.

Activation of Akt cascade is pivotal in mediating survival response in neuronal cells [69, 70]. A number of different agents (or stimuli) could activate Akt cascade to inhibit neuronal cell injury and apoptosis by ischemia-reperfusion or OGD/R [71–77]. These agents include heat shock protein B8 (HspB8) [76], curcumin [73], baicalein [74], humanin [75], and several neurotrophic factors [69, 70]. Conversely, Akt blockage, mainly through Akt inhibitors, reversed the neuroprotective effect by the agents [72–76]. We provided evidence to support that Akt activation was required for NLGN3-induced neuroprotection. Blockage of Akt activation, by MK-2206 or Akt1/2 shRNA, completely reversed NLGN3-induced neuronal cytoprotection against OGD/R. Conversely, forced activation of Akt by caAkt1 mimicked NLGN3-induced actions and ameliorated OGD/R-induced neuronal cell death. Significantly, NLGN3 failed to offer additional neuroprotection against OGD/R in caAkt1-expressing SH-SY5Y cells. Therefore, Akt activation was the key mechanism of NLGN3-induced neuroprotection against OGD/R.

Importantly, ADAM10 inhibition by lateral ventricle injection of GI254023X blocked MCAO-induced NLGN3 cleavage and secretion, inhibited Akt activation and exacerbated ischemic brain injury in mice. Moreover, neuronal silencing of NLGN3, by lateral ventricle injection of shRNA adenovirus, inhibited Akt activation and exacerbated MCAO-induced ischemic brain injury. Conversely, neuronal overexpression of NLGN3 increased Akt activation and alleviated MCAO-induced ischemic brain injury in mice.

Our previous studies have proposed the pivotal role of Gαi1/3 proteins in mediating RTKs-induced signalings. Sun et al., have shown that Gαi1/3 mediated VEGF-induced signaling [34]. Gαi1/3 are in the VEGFR2 endocytosis complex (VEGFR2-Ephrin-B2-Dab2-PAR-3), required for VEGF-induced VEGFR2 endocytosis and downstream signaling activation [34]. Gαi1/3 are also key signaling proteins for brain-derived neurotrophic factor (BDNF)-induced signaling in neurons [33]. In mouse embryonic fibroblasts (MEFs) and neurons, Gαi1/3 silencing or KO largely inhibited BDNF-induced downstream signaling (Akt-mTOR and Erk-MAPK) activation [33]. Similarly, Gαi1/3 are indispensable for keratinocyte growth factor (KGF)- and epidermal growth factor (EGF)-induced Akt-mTOR signaling [30, 32].

We here found that Gαi1/3 should be key proteins mediating NLGN3-induced Akt signaling in neuronal cells. In SH-SY5Y cells and primary murine neurons, shRNA/siRNA-induced silencing or CRISPR/Cas9-induced KO of Gαi1/3 largely inhibited NLGN3-induced Akt-S6K1 activation. Importantly, Gαi1/3 silencing or depletion almost abolished NLGN3-induced neuronal cytoprotection against OGD/R. Significantly, Gαi1/3 neuronal silencing, by lateral ventricle injection of shRNA adenovirus, inhibited Akt activation and exacerbated MCAO-induced ischemic brain injury. Thus, Gαi1/3-mediating Akt activation is required for NLGN3-induced neuroprotection against ischemia-reperfusion injury.

## Conclusion

NLGN3 protects neuronal cells from ischemia-reperfusion injury via activation of Gαi1/3-Akt signaling.

### Reporting summary

Further information on research design is available in the Nature Research Reporting Summary linked to this article.

### Supplementary information

Original Data File

Figure S1

Reporting Summary

Author contribution FORM

## Data Availability

The data are included in the article.

## References

[1] Shahjouei S, Sadighi A, Chaudhary D, Li J, Abedi V, Holland N (2021). A 5-decade analysis of incidence trends of ischemic stroke after transient ischemic attack: a systematic review and meta-analysis. JAMA Neurol.

[2] Mendelson SJ, Prabhakaran S (2021). Diagnosis and management of transient ischemic attack and acute ischemic stroke: a review. JAMA.

[3] Powers WJ, Rabinstein AA, Ackerson T, Adeoye OM, Bambakidis NC, Becker K (2019). Guidelines for the early management of patients with acute ischemic stroke: 2019 update to the 2018 Guidelines for the Early Management of Acute Ischemic Stroke: A Guideline for Healthcare Professionals From the American Heart Association/American Stroke Association. Stroke.

[4] Fuentes B, Tejedor ED (2014). Stroke: the worldwide burden of stroke-a blurred photograph. Nat Rev Neurol.

[5] Tymianski M (2014). Stroke in 2013: disappointments and advances in acute stroke intervention. Nat Rev Neurol.

[6] Clark WM, Clark TD (2012). Stroke: treatment for acute stroke-the end of the citicoline saga. Nat Rev Neurol.

[7] Planas AM (2013). Advances in stroke: translational medicine 2012. Stroke.

[8] Verklan MT (2009). The chilling details: hypoxic-ischemic encephalopathy. J Perinat Neonatal Nurs.

[9] Allen CL, Bayraktutan U (2009). Oxidative stress and its role in the pathogenesis of ischaemic stroke. Int J Stroke.

[10] Xu S, Li Y, Chen JP, Li DZ, Jiang Q, Wu T (2020). Oxygen glucose deprivation/re-oxygenation-induced neuronal cell death is associated with Lnc-D63785 m6A methylation and miR-422a accumulation. Cell Death Dis.

[11] Zhao LP, Ji C, Lu PH, Li C, Xu B, Gao H (2013). Oxygen glucose deprivation (OGD)/re-oxygenation-induced in vitro neuronal cell death involves mitochondrial cyclophilin-D/P53 signaling axis. Neurochem Res.

[12] Gu DM, Lu PH, Zhang K, Wang X, Sun M, Chen GQ (2015). EGFR mediates astragaloside IV-induced Nrf2 activation to protect cortical neurons against in vitro ischemia/reperfusion damages. Biochem Biophys Res Commun.

[13] Almeida A, Delgado-Esteban M, Bolanos JP, Medina JM (2002). Oxygen and glucose deprivation induces mitochondrial dysfunction and oxidative stress in neurones but not in astrocytes in primary culture. J Neurochem.

[14] Zhao H, Mitchell S, Ciechanowicz S, Savage S, Wang T, Ji X (2016). Argon protects against hypoxic-ischemic brain injury in neonatal rats through activation of nuclear factor (erythroid-derived 2)-like 2. Oncotarget.

[15] Foldy C, Malenka RC, Sudhof TC (2013). Autism-associated neuroligin-3 mutations commonly disrupt tonic endocannabinoid signaling. Neuron.

[16] Budreck EC, Scheiffele P (2007). Neuroligin-3 is a neuronal adhesion protein at GABAergic and glutamatergic synapses. Eur J Neurosci.

[17] Yan J, Oliveira G, Coutinho A, Yang C, Feng J, Katz C (2005). Analysis of the neuroligin 3 and 4 genes in autism and other neuropsychiatric patients. Mol Psychiatry.

[18] Venkatesh HS, Johung TB, Caretti V, Noll A, Tang Y, Nagaraja S (2015). Neuronal activity promotes glioma growth through neuroligin-3 secretion. Cell.

[19] Venkatesh HS, Tam LT, Woo PJ, Lennon J, Nagaraja S, Gillespie SM (2017). Targeting neuronal activity-regulated neuroligin-3 dependency in high-grade glioma. Nature.

[20] Thompson EG, Sontheimer H (2015). A frightening thought: neuronal activity enhances tumor growth. Cell Res.

[21] Johung T, Monje M (2017). Neuronal activity in the glioma microenvironment. Curr Opin Neurobiol.

[22] Lin L, Wang Q, Qian K, Cao Z, Xiao J, Wang X (2018). bFGF protects against oxygen glucose deprivation/reoxygenation-induced endothelial monolayer permeability via S1PR1-dependent mechanisms. Mol Neurobiol.

[23] Xiong S, Xu Y, Ma M, Wang H, Wei F, Gu Q (2017). Neuroprotective effects of a novel peptide, FK18, under oxygen-glucose deprivation in SH-SY5Y cells and retinal ischemia in rats via the Akt pathway. Neurochem Int.

[24] Lemarchand E, Maubert E, Haelewyn B, Ali C, Rubio M, Vivien D (2016). Stressed neurons protect themselves by a tissue-type plasminogen activator-mediated EGFR-dependent mechanism. Cell Death Differ.

[25] Mielke JG, Taghibiglou C, Wang YT (2006). Endogenous insulin signaling protects cultured neurons from oxygen-glucose deprivation-induced cell death. Neuroscience.

[26] Wang DP, Jin KY, Zhao P, Lin Q, Kang K, Hai J (2021). Neuroprotective effects of VEGF-A nanofiber membrane and FAAH inhibitor URB597 against oxygen-glucose deprivation-induced ischemic neuronal injury. Int J Nanomed.

[27] Duarte EP, Curcio M, Canzoniero LM, Duarte CB (2012). Neuroprotection by GDNF in the ischemic brain. Growth Factors.

[28] Hennigan A, O’Callaghan RM, Kelly AM (2007). Neurotrophins and their receptors: roles in plasticity, neurodegeneration and neuroprotection. Biochem Soc Trans.

[29] Alessi DR, James SR, Downes CP, Holmes AB, Gaffney PR, Reese CB (1997). Characterization of a 3-phosphoinositide-dependent protein kinase which phosphorylates and activates protein kinase Balpha. Curr Biol.

[30] Cao C, Huang X, Han Y, Wan Y, Birnbaumer L, Feng GS (2009). Galpha(i1) and Galpha(i3) are required for epidermal growth factor-mediated activation of the Akt-mTORC1 pathway. Sci Signal.

[31] Liu YY, Chen MB, Cheng L, Zhang ZQ, Yu ZQ, Jiang Q (2018). microRNA-200a downregulation in human glioma leads to Galphai1 over-expression, Akt activation, and cell proliferation. Oncogene.

[32] Zhang YM, Zhang ZQ, Liu YY, Zhou X, Shi XH, Jiang Q (2015). Requirement of Galphai1/3-Gab1 signaling complex for keratinocyte growth factor-induced PI3K-AKT-mTORC1 activation. J Invest Dermatol.

[33] Marshall J, Zhou XZ, Chen G, Yang SQ, Li Y, Wang Y (2018). Antidepression action of BDNF requires and is mimicked by Galphai1/3 expression in the hippocampus. Proc Natl Acad Sci USA.

[34] Sun J, Huang W, Yang SF, Zhang XP, Yu Q, Zhang ZQ (2018). Galphai1 and Galphai3mediate VEGF-induced VEGFR2 endocytosis, signaling and angiogenesis. Theranostics.

[35] Zheng Y, Chen Z, She C, Lin Y, Hong Y, Shi L (2020). Four-octyl itaconate activates Nrf2 cascade to protect osteoblasts from hydrogen peroxide-induced oxidative injury. Cell Death Dis.

[36] Wang Y, Liu YY, Chen MB, Cheng KW, Qi LN, Zhang ZQ (2021). Neuronal-driven glioma growth requires Galphai1 and Galphai3. Theranostics.

[37] Shan HJ, Zhu LQ, Yao C, Zhang ZQ, Liu YY, Jiang Q (2021). MAFG-driven osteosarcoma cell progression is inhibited by a novel miRNA miR-4660. Mol Ther Nucleic Acids.

[38] Bai JY, Li Y, Xue GH, Li KR, Zheng YF, Zhang ZQ (2021). Requirement of Galphai1 and Galphai3 in interleukin-4-induced signaling, macrophage M2 polarization and allergic asthma response. Theranostics.

[39] Cao C, Rioult-Pedotti MS, Migani P, Yu CJ, Tiwari R, Parang K (2013). Impairment of TrkB-PSD-95 signaling in Angelman syndrome. PLoS Biol.

[40] Liu H, Feng Y, Xu M, Yang J, Wang Z, Di G (2018). Four-octyl itaconate activates Keap1-Nrf2 signaling to protect neuronal cells from hydrogen peroxide. Cell Commun Signal.

[41] Liu H, Zhang Z, Xu M, Xu R, Wang Z, Di G (2018). K6PC-5 activates SphK1-Nrf2 signaling to protect neuronal cells from oxygen glucose deprivation/re-oxygenation. Cell Physiol Biochem.

[42] Di G, Wang Z, Wang W, Cheng F, Liu H (2017). AntagomiR-613 protects neuronal cells from oxygen glucose deprivation/re-oxygenation via increasing SphK2 expression. Biochem Biophys Res Commun.

[43] Wang TB, Geng M, Jin H, Tang AG, Sun H, Zhou LZ (2021). SREBP1 site 1 protease inhibitor PF-429242 suppresses renal cell carcinoma cell growth. Cell Death Dis.

[44] Brooks MM, Neelam S, Fudala R, Gryczynski I, Cammarata PR (2013). Lenticular mitoprotection. Part A: monitoring mitochondrial depolarization with JC-1 and artifactual fluorescence by the glycogen synthase kinase-3beta inhibitor, SB216763. Mol Vis.

[45] Zheng K, Sheng Z, Li Y, Lu H (2014). Salidroside inhibits oxygen glucose deprivation (OGD)/re-oxygenation-induced H9c2 cell necrosis through activating of Akt-Nrf2 signaling. Biochem Biophys Res Commun.

[46] Qin LS, Jia PF, Zhang ZQ, Zhang SM (2015). ROS-p53-cyclophilin-D signaling mediates salinomycin-induced glioma cell necrosis. J Exp Clin Cancer Res.

[47] Li C, Yan K, Wang W, Bai Q, Dai C, Li X (2017). MIND4-17 protects retinal pigment epithelium cells and retinal ganglion cells from UV. Oncotarget.

[48] Wen H, Li L, Zhan L, Zuo Y, Li K, Qiu M (2021). Hypoxic postconditioning promotes mitophagy against transient global cerebral ischemia via PINK1/Parkin-induced mitochondrial ubiquitination in adult rats. Cell Death Dis.

[49] Liu TT, Shi X, Hu HW, Chen JP, Jiang Q, Zhen YF (2023). Endothelial cell-derived RSPO3 activates Galphai1/3-Erk signaling and protects neurons from ischemia/reperfusion injury. Cell Death Dis.

[50] Li XM, Huang D, Yu Q, Yang J, Yao J (2018). Neuroligin-3 protects retinal cells from H(2)O(2)-induced cell death via activation of Nrf2 signaling. Biochem Biophys Res Commun.

[51] Weng Y, Lin J, Liu H, Wu H, Yan Z, Zhao J (2018). AMPK activation by Tanshinone IIA protects neuronal cells from oxygen-glucose deprivation. Oncotarget.

[52] Wang M, Jiang YM, Xia LY, Wang Y, Li WY, Jin T (2018). LncRNA NKILA upregulation mediates oxygen glucose deprivation/re-oxygenation-induced neuronal cell death by inhibiting NF-kappaB signaling. Biochem Biophys Res Commun.

[53] Zheng K, Zhang Q, Sheng Z, Li Y, Lu HH (2018). Ciliary neurotrophic factor (CNTF) protects myocardial cells from oxygen glucose deprivation (OGD)/re-oxygenation via activation of Akt-Nrf2 signaling. Cell Physiol Biochem.

[54] Yang X, He XQ, Li GD, Xu YQ (2017). AntagomiR-451 inhibits oxygen glucose deprivation (OGD)-induced HUVEC necrosis via activating AMPK signaling. PLoS ONE.

[55] Tang XF, Liu HY, Wu L, Li MH, Li SP, Xu HB (2017). Ginseng Rh2 protects endometrial cells from oxygen glucose deprivation/re-oxygenation. Oncotarget.

[56] Song G, Ouyang G, Bao S (2005). The activation of Akt/PKB signaling pathway and cell survival. J Cell Mol Med.

[57] Zhang Y, Gong XG, Wang ZZ, Sun HM, Guo ZY, Hu JH (2016). Overexpression of DJ-1/PARK7, the Parkinson’s disease-related protein, improves mitochondrial function via Akt phosphorylation on threonine 308 in dopaminergic neuron-like cells. Eur J Neurosci.

[58] Morgan-Warren PJ, Berry M, Ahmed Z, Scott RA, Logan A (2013). Exploiting mTOR signaling: a novel translatable treatment strategy for traumatic optic neuropathy?. Invest Ophthalmol Vis Sci.

[59] Lin DC, Quevedo C, Brewer NE, Bell A, Testa JR, Grimes ML (2006). APPL1 associates with TrkA and GIPC1 and is required for nerve growth factor-mediated signal transduction. Mol Cell Biol.

[60] Kaplan DR, Miller FD (2000). Neurotrophin signal transduction in the nervous system. Curr Opin Neurobiol.

[61] Ji D, Zhang Z, Cheng L, Chang J, Wang S, Zheng B (2014). The combination of RAD001 and MK-2206 exerts synergistic cytotoxic effects against PTEN mutant gastric cancer cells: involvement of MAPK-dependent autophagic, but not apoptotic cell death pathway. PLoS ONE.

[62] Yap TA, Yan L, Patnaik A, Fearen I, Olmos D, Papadopoulos K (2011). First-in-man clinical trial of the oral pan-AKT inhibitor MK-2206 in patients with advanced solid tumors. J Clin Oncol.

[63] Hirai H, Sootome H, Nakatsuru Y, Miyama K, Taguchi S, Tsujioka K (2010). MK-2206, an allosteric Akt inhibitor, enhances antitumor efficacy by standard chemotherapeutic agents or molecular targeted drugs in vitro and in vivo. Mol Cancer Ther.

[64] Yang H, Zhao J, Zhao M, Zhao L, Zhou LN, Duan Y (2020). GDC-0349 inhibits non-small cell lung cancer cell growth. Cell Death Dis.

[65] Venkataramani V, Tanev DI, Strahle C, Studier-Fischer A, Fankhauser L, Kessler T (2019). Glutamatergic synaptic input to glioma cells drives brain tumour progression. Nature.

[66] Venkatesh HS, Morishita W, Geraghty AC, Silverbush D, Gillespie SM, Arzt M (2019). Electrical and synaptic integration of glioma into neural circuits. Nature.

[67] Xu HB, Zheng YF, Wu D, Li Y, Zhou LN, Chen YG (2020). microRNA-1203 targets and silences cyclophilin D to protect human endometrial cells from oxygen and glucose deprivation-re-oxygenation. Aging (Albany NY).

[68] Zheng K, Zhang Q, Lin G, Li Y, Sheng Z, Wang J (2017). Activation of Akt by SC79 protects myocardiocytes from oxygen and glucose deprivation (OGD)/re-oxygenation. Oncotarget.

[69] Ahn JY (2014). Neuroprotection signaling of nuclear akt in neuronal cells. Exp Neurobiol.

[70] Brunet A, Datta SR, Greenberg ME (2001). Transcription-dependent and -independent control of neuronal survival by the PI3K-Akt signaling pathway. Curr Opin Neurobiol.

[71] Gao G, Fan H, Zhang X, Zhang F, Wu H, Qi F (2017). Neuroprotective effect of G(14)-humanin on global cerebral ischemia/reperfusion by activation of SOCS3 - STAT3 - MCL-1 signal transduction pathway in rats. Neurol Res.

[72] Ramirez-Sanchez J, Simoes Pires EN, Nunez-Figueredo Y, Pardo-Andreu GL, Fonseca-Fonseca LA, Ruiz-Reyes A (2015). Neuroprotection by JM-20 against oxygen-glucose deprivation in rat hippocampal slices: Involvement of the Akt/GSK-3beta pathway. Neurochem Int.

[73] Wu J, Li Q, Wang X, Yu S, Li L, Wu X (2013). Neuroprotection by curcumin in ischemic brain injury involves the Akt/Nrf2 pathway. PLoS ONE.

[74] Liu C, Wu J, Xu K, Cai F, Gu J, Ma L (2010). Neuroprotection by baicalein in ischemic brain injury involves PTEN/AKT pathway. J Neurochem.

[75] Xu X, Chua CC, Gao J, Chua KW, Wang H, Hamdy RC (2008). Neuroprotective effect of humanin on cerebral ischemia/reperfusion injury is mediated by a PI3K/Akt pathway. Brain Res.

[76] Hu Z, Yang B, Mo X, Zhou F (2016). HspB8 mediates neuroprotection against OGD/R in N2A cells through the phosphoinositide 3-kinase/Akt pathway. Brain Res.

[77] Rau TF, Kothiwal A, Zhang L, Ulatowski S, Jacobson S, Brooks DM (2011). Low dose methamphetamine mediates neuroprotection through a PI3K-AKT pathway. Neuropharmacology.

