# Supplementary material for: Neuron-secreted NLGN3 ameliorates ischemic brain injury via activating Gαi1/3-Akt signaling
Source: Cell Death Dis. 2023 Oct 25;14(10):700. doi: 10.1038/s41419-023-06219-8 (PMC10600254; doi:10.1038/s41419-023-06219-8)

Figure S2: The uncropped blotting images of the study.

Figure 1

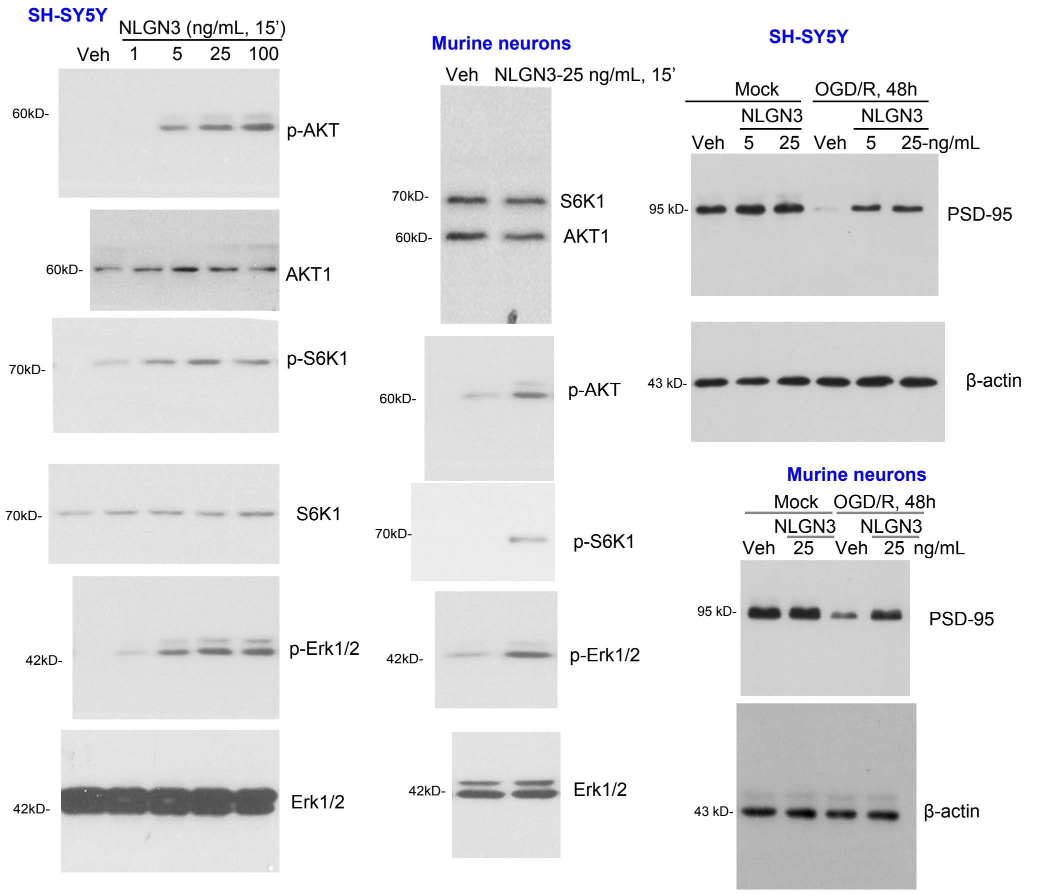

Figure 2

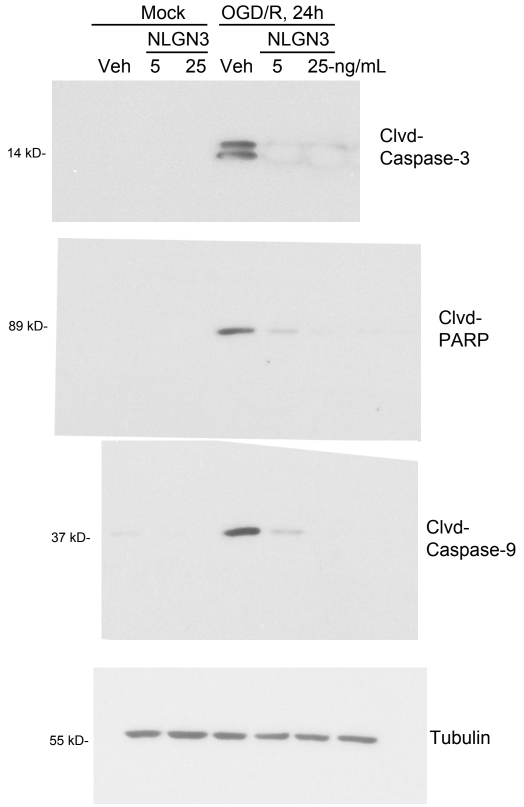

Figure 3.

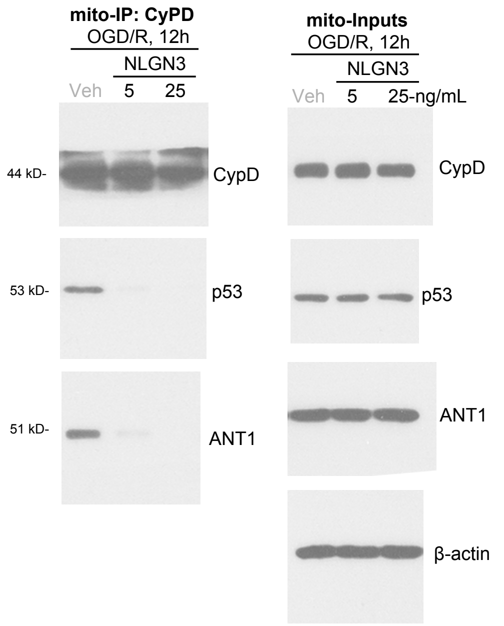

Figure 4.

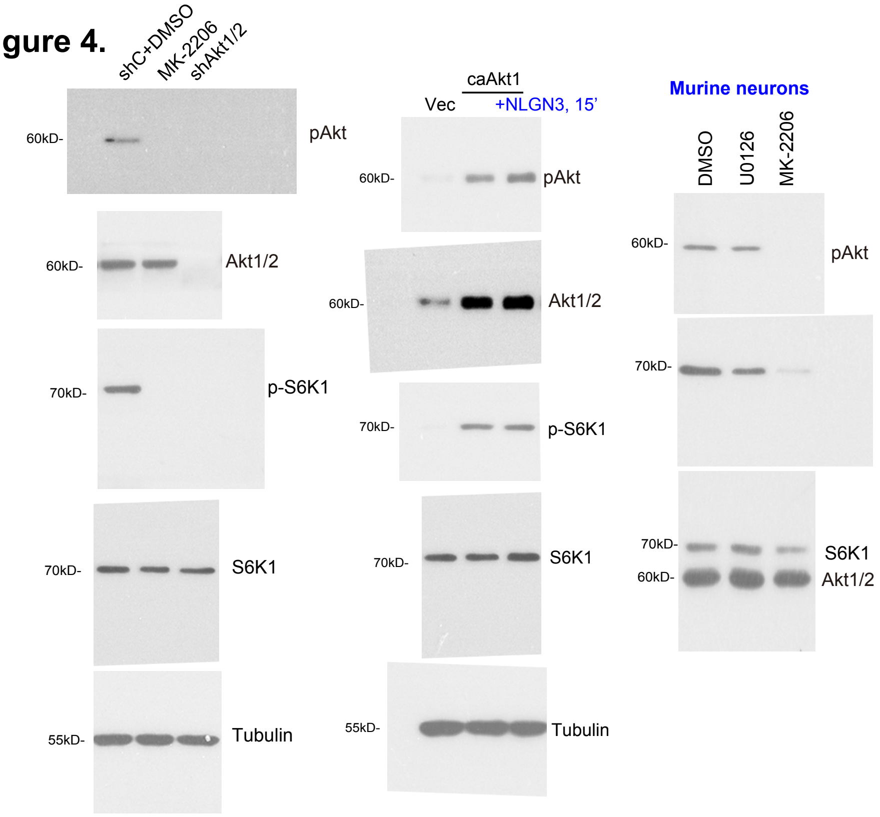

Figure 5

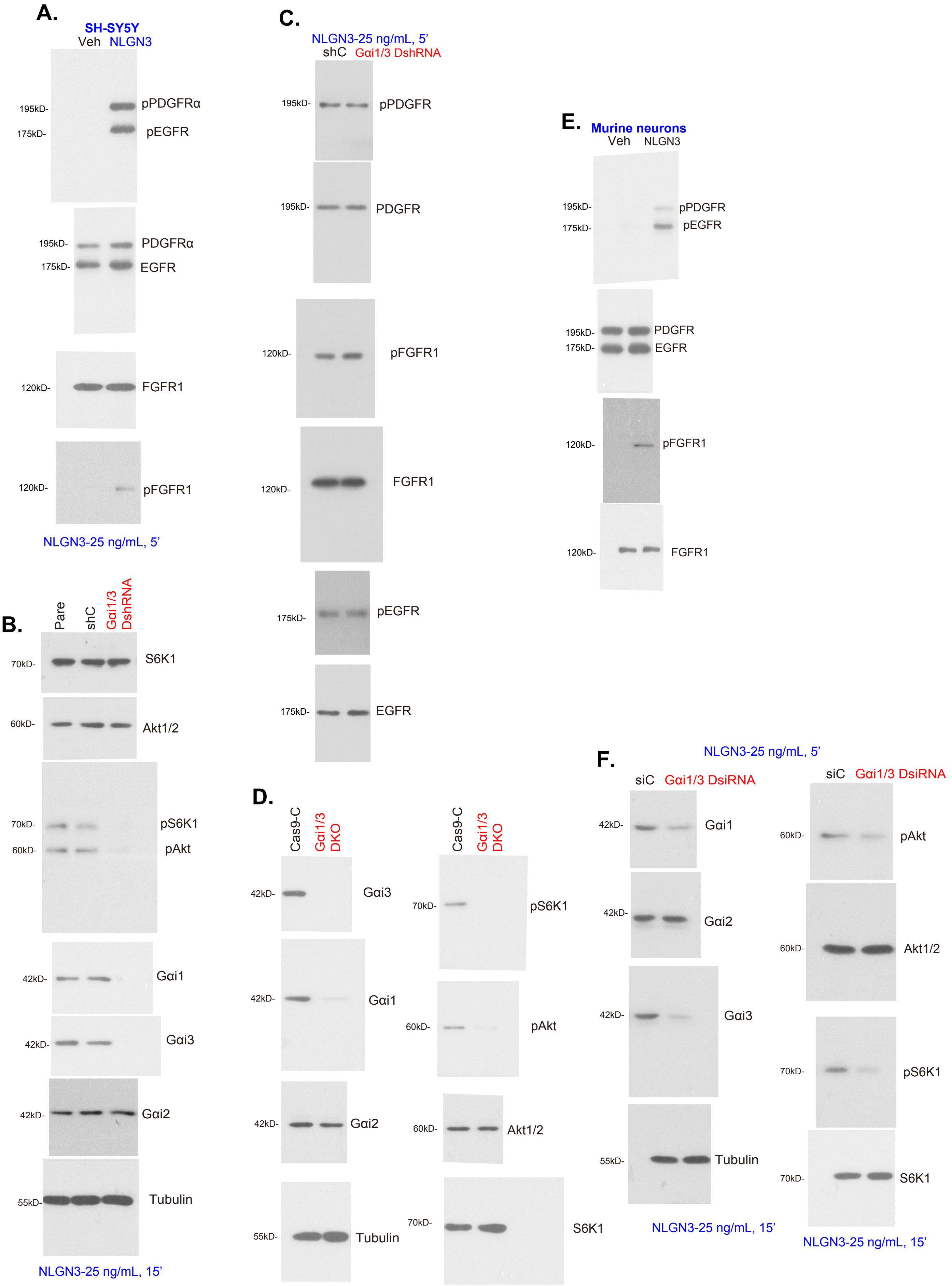

A.
SH-SY5Y
Veh NLGN3
195kD- pPDGFRα
175kD- pEGFR
195kD- PDGFRα
175kD- EGFR
120kD- FGFR1
120kD- pFGFR1
NLGN3-25 ng/mL, 5'
B.
Pare shC Gαi1/3 DshRNA
70kD- S6K1
60kD- Akt1/2
70kD- pS6K1
60kD- pAkt
42kD- Gαi1
42kD- Gαi3
42kD- Gαi2
55kD- Tubulin
NLGN3-25 ng/mL, 15'
C.
NLGN3-25 ng/mL, 5'
shC Gαi1/3 DshRNA
195kD- pPDGFR
195kD- PDGFR
120kD- pFGFR1
120kD- FGFR1
175kD- pEGFR
175kD- EGFR
D.
Cas9-C Gαi1/3 DKO
42kD- Gαi3
42kD- Gαi1
42kD- Gαi2
55kD- Tubulin
Cas9-C Gαi1/3 DKO
70kD- pS6K1
60kD- pAkt
60kD- Akt1/2
70kD- S6K1
E.
Murine neurons
Veh NLGN3
195kD- pPDGFR
175kD- pEGFR
195kD- PDGFR
175kD- EGFR
120kD- pFGFR1
120kD- FGFR1
F.
NLGN3-25 ng/mL, 5'
siC Gαi1/3 DsiRNA
42kD- Gαi1
42kD- Gαi2
42kD- Gαi3
55kD- Tubulin
NLGN3-25 ng/mL, 15'
siC Gαi1/3 DsiRNA
60kD- pAkt
60kD- Akt1/2
70kD- pS6K1
70kD- S6K1
NLGN3-25 ng/mL, 15'

Figure 7.

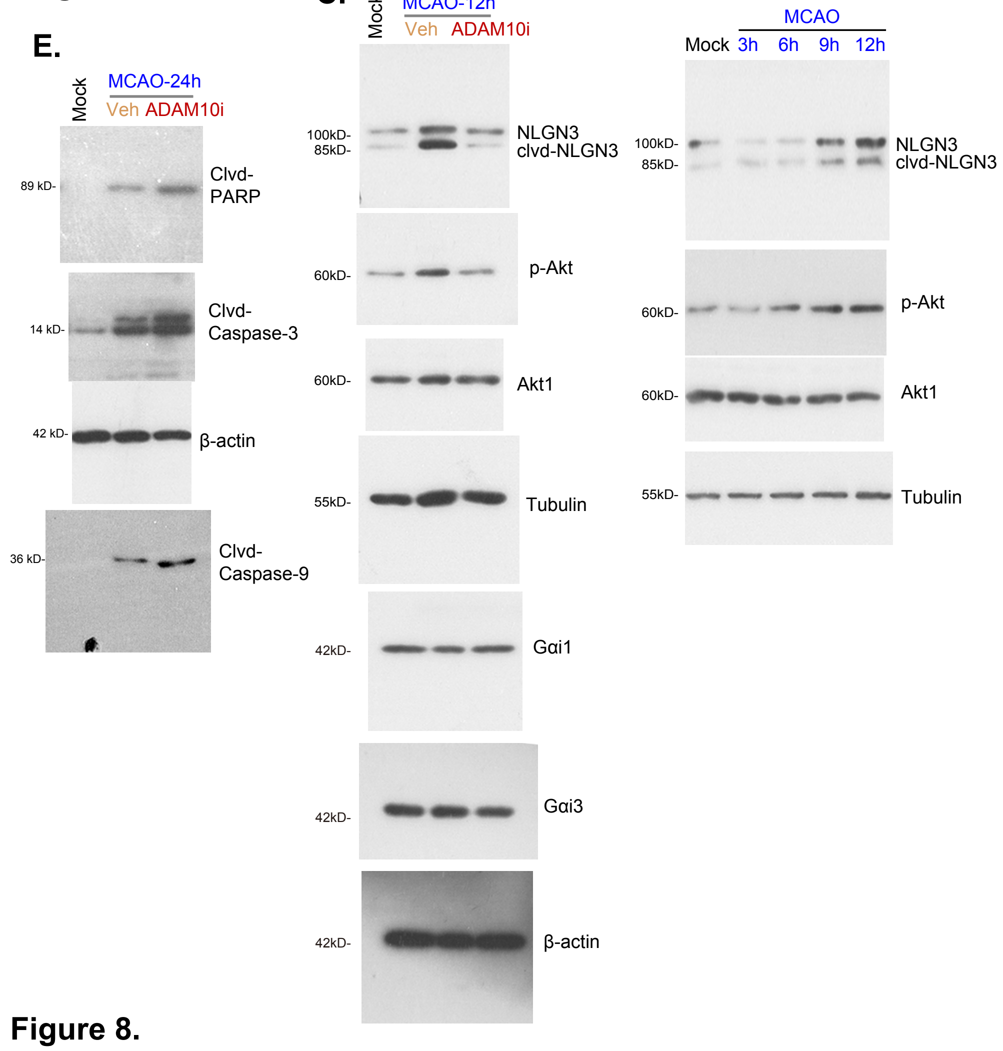

Figure 9.

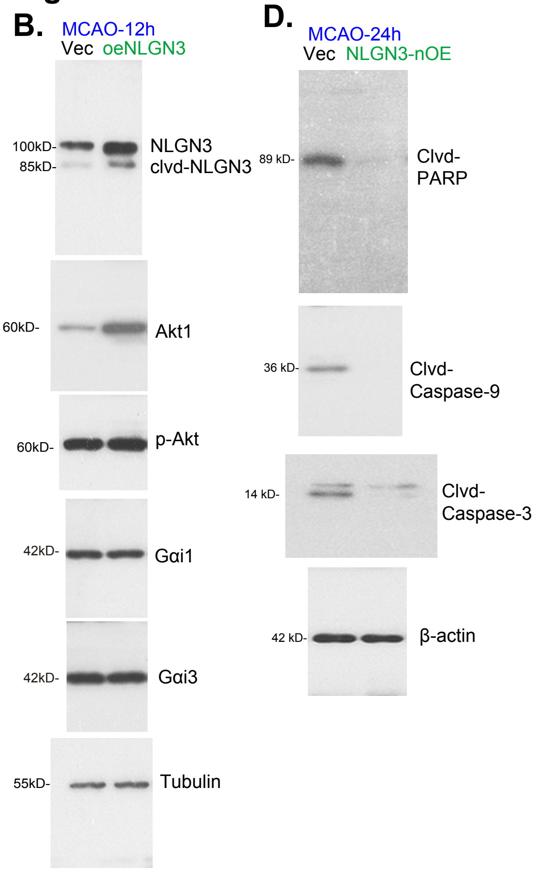

Figure 8.

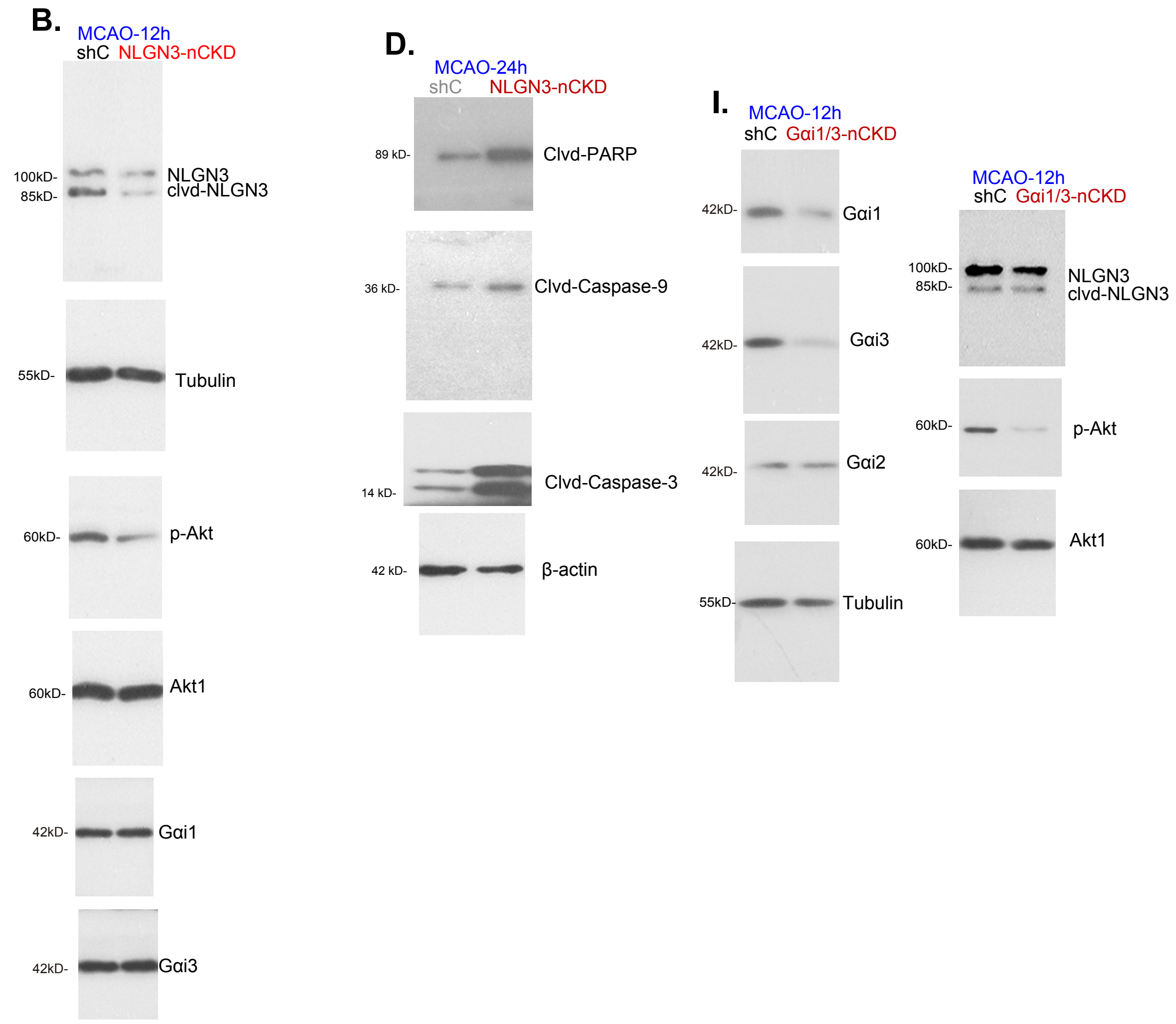

Supplement: Supplementary file 1 — Original Data File [file 41419_2023_6219_MOESM1_ESM.pdf]
