# Supplementary material for: Neuron-secreted NLGN3 ameliorates ischemic brain injury via activating Gαi1/3-Akt signaling
Source: Cell Death Dis. 2023 Oct 25;14(10):700. doi: 10.1038/s41419-023-06219-8 (PMC10600254; doi:10.1038/s41419-023-06219-8)

Figure S1.

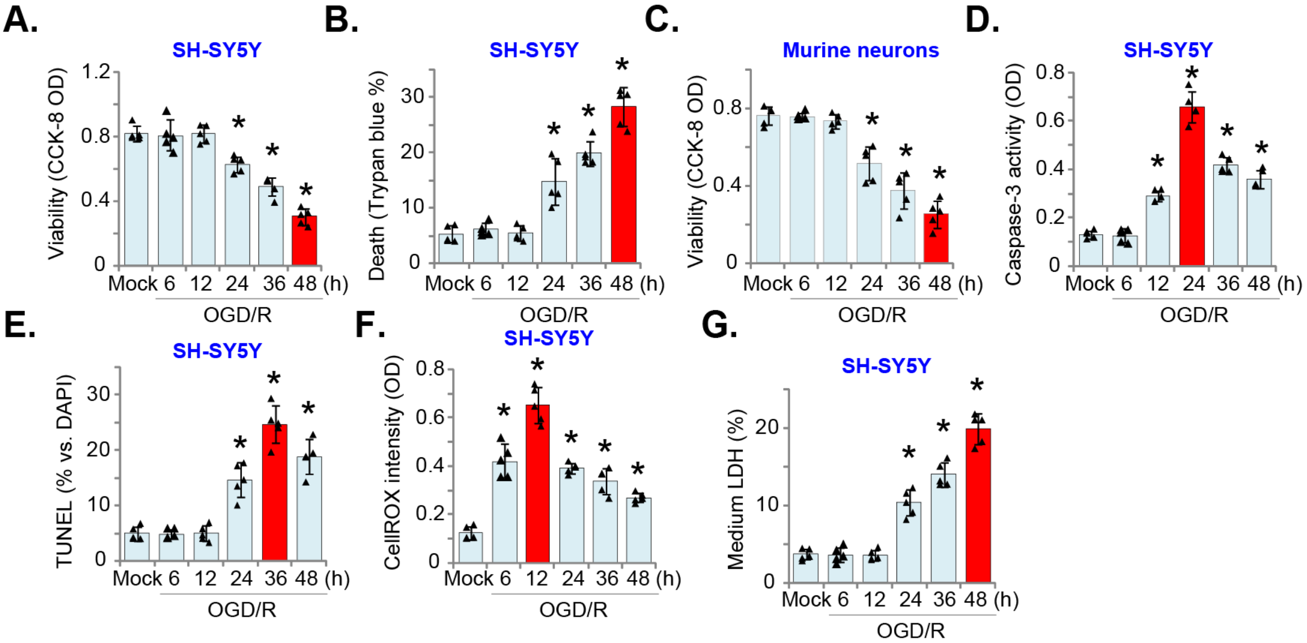

Figure S1. OGD/R induces a time-dependent response in neuronal cells. The differentiated SH-SY5Y neuronal cells or the primary murine cortical neurons were maintained under oxygen glucose deprivation (OGD) for 4h and then re-oxygenation ("OGD/R") for the applied time periods; Cell viability (CCK-8 OD, **A** and **C**), cell death (Trypan blue staining, **B**), Caspase-3 activity (**D**) as well as cell apoptosis (by measuring TUNEL-positive nuclei ratio, **E**), ROS production (via measuring CellROX intensity, **F**) and cell necrosis (by measuring medium LDH release, **G**) were tested. The red bar stands for the most significant time point in each assay. "Mock" stands for the mock treatment (norm-oxygenated medium with glucose). Data were presented as mean ± standard deviation (SD, n=5). * ***P*** <0.05 *vs.* "Mock" cells. Each experiment was repeated five times and similar results were obtained.

Figure S2: The uncropped blotting images of the study.

Figure 1

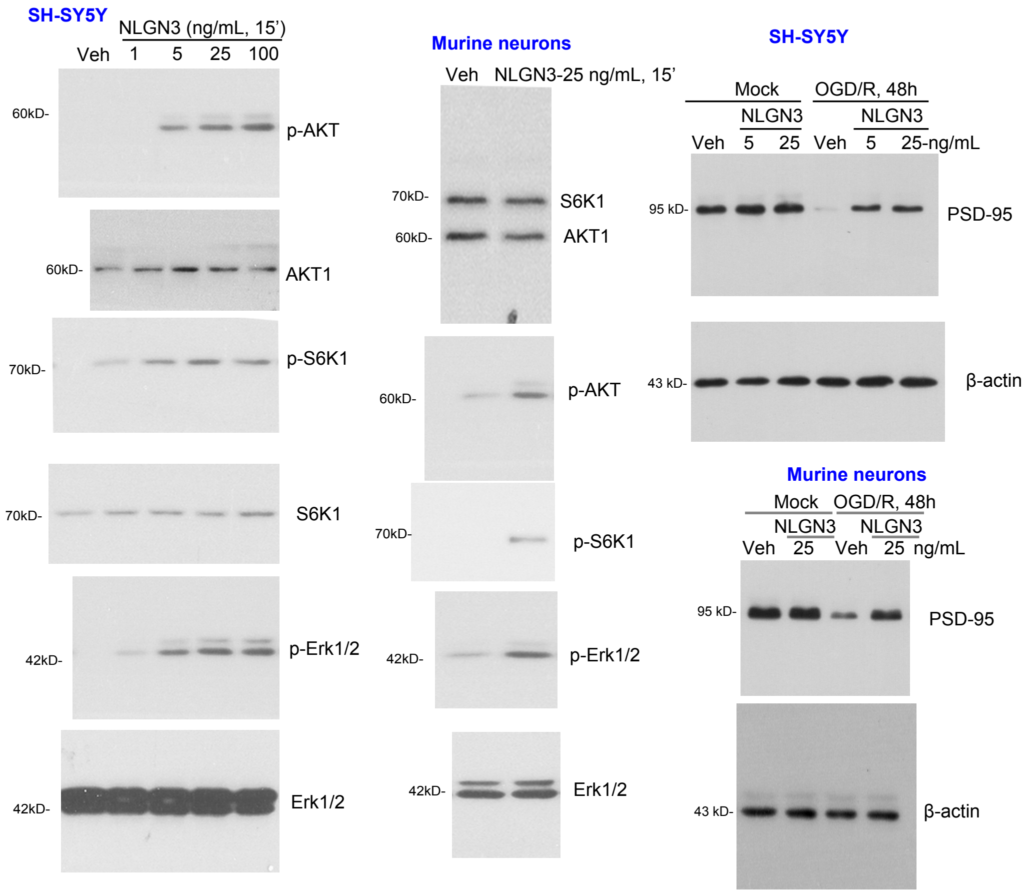

Figure 2

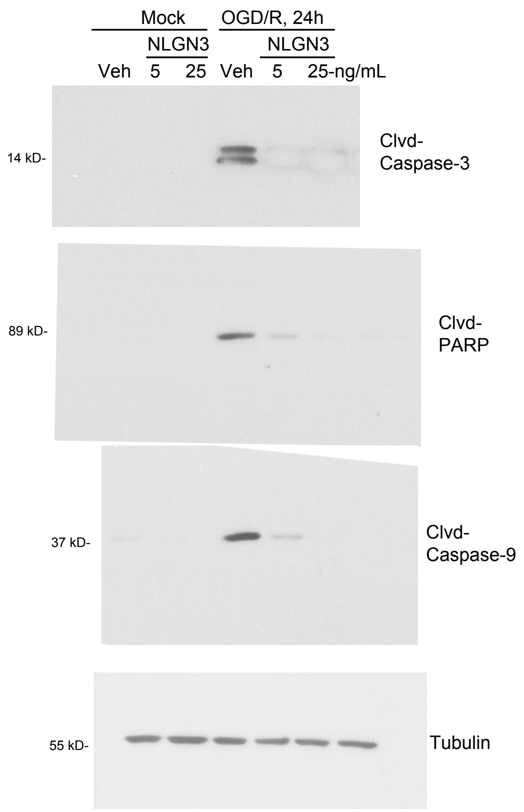

Figure 3.

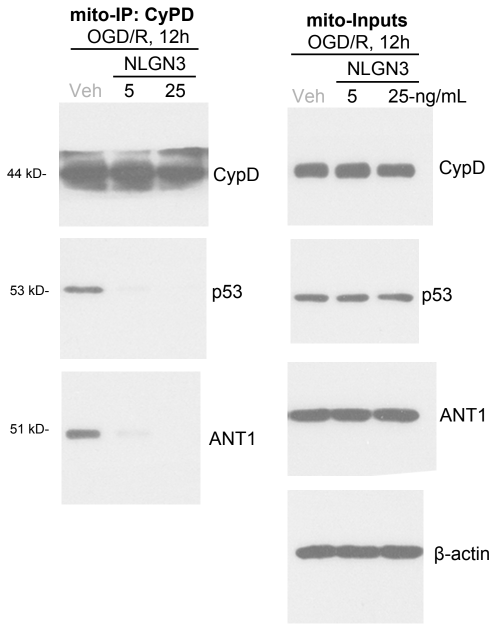

Figure 4.

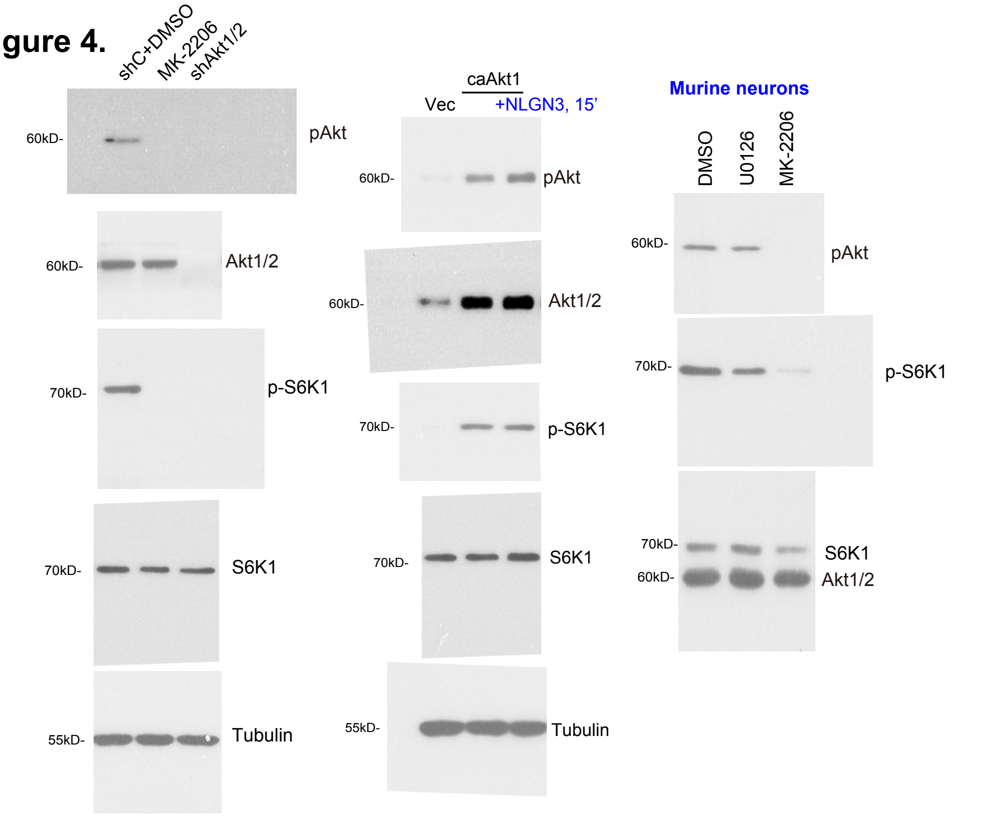

Figure 5

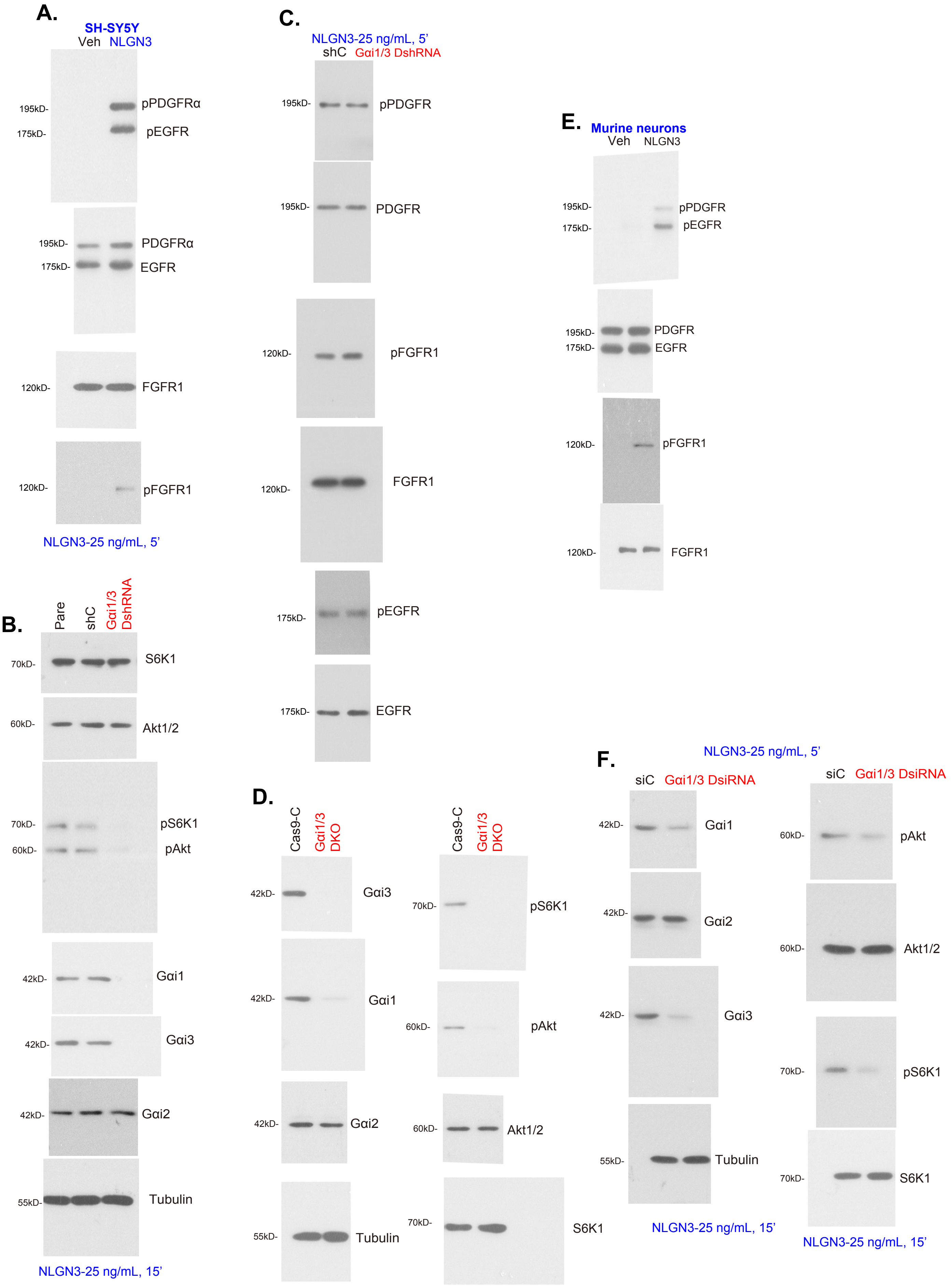
A.
SH-SY5Y
Veh NLGN3
195kD-
175kD-
pPDGFRα
pEGFR
PDGFRα
EGFR
120kD-
FGFR1
pFGFR1
NLGN3-25 ng/mL, 5'
B.
Pare
shC
Gαi1/3 DshRNA
70kD-
S6K1
60kD-
Akt1/2
pS6K1
pAkt
42kD-
Gαi1
Gαi3
Gαi2
55kD-
Tubulin
NLGN3-25 ng/mL, 15'
C.
NLGN3-25 ng/mL, 5'
shC Gαi1/3 DshRNA
pPDGFR
PDGFR
pFGFR1
FGFR1
pEGFR
EGFR
D.
Cas9-C
Gαi1/3 DKO
Gαi3
Gαi1
Gαi2
Tubulin
pS6K1
pAkt
Akt1/2
S6K1
E.
Murine neurons
Veh NLGN3
pPDGFR
pEGFR
PDGFR
EGFR
pFGFR1
FGFR1
F.
NLGN3-25 ng/mL, 5'
siC Gαi1/3 DsiRNA
Gαi1
Gαi2
Gαi3
Tubulin
NLGN3-25 ng/mL, 15'
pAkt
Akt1/2
pS6K1
S6K1

Figure 7.

E.

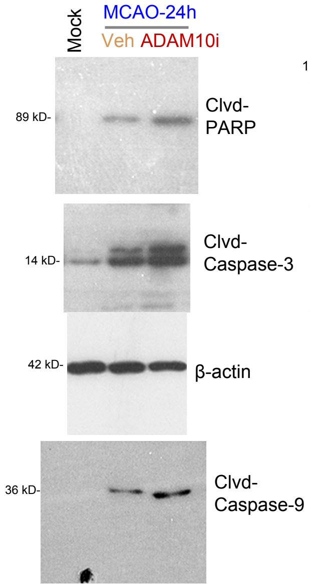

C.

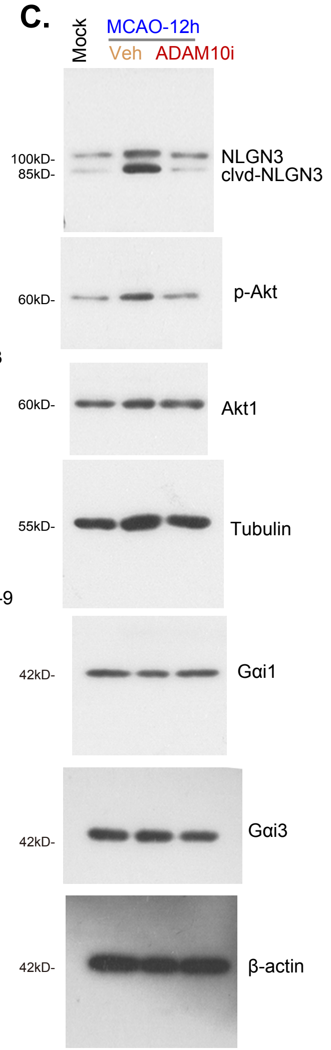

Figure 9.

A.

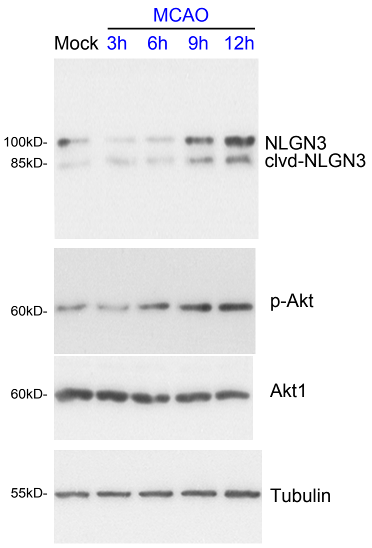

B.

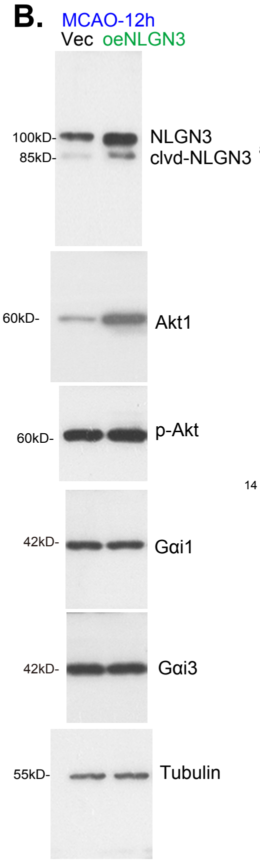

D.

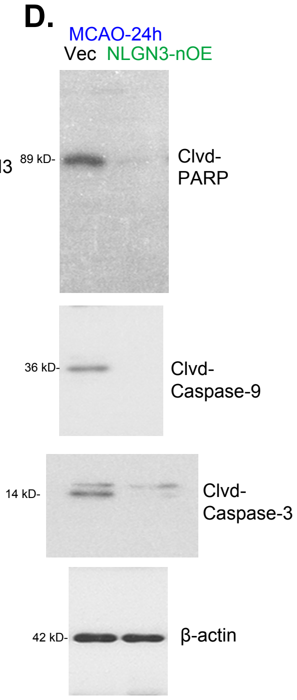

Figure 8.

B.

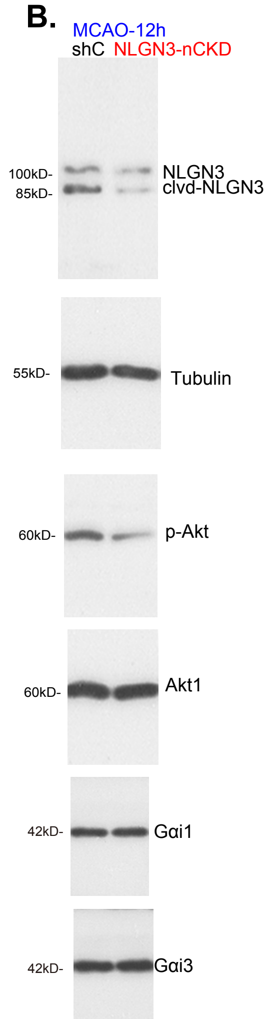

D.

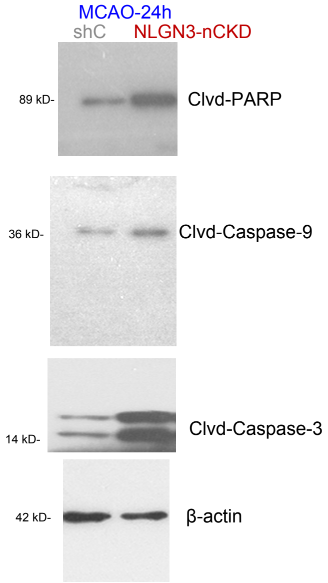

I.

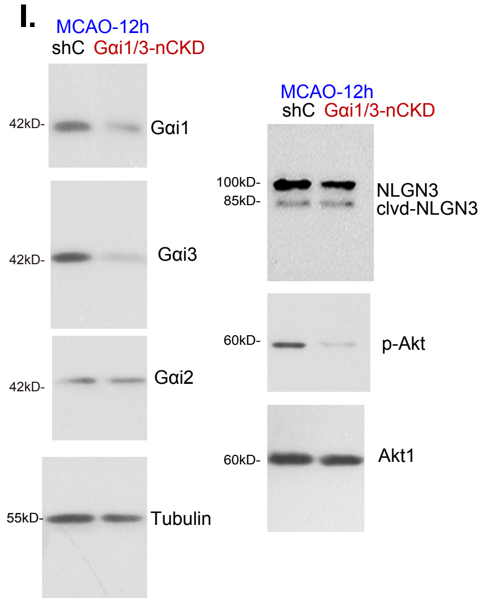

Supplement: Supplementary file 2 — Figure S1 [file 41419_2023_6219_MOESM2_ESM.pdf]
