# Supplementary material for: Neuron-secreted NLGN3 ameliorates ischemic brain injury via activating Gαi1/3-Akt signaling
Source: Cell Death Dis. 2023 Oct 25;14(10):700. doi: 10.1038/s41419-023-06219-8 (PMC10600254; doi:10.1038/s41419-023-06219-8)
Supplement: Supplementary file 4 — Author contribution FORM [file 41419_2023_6219_MOESM4_ESM.pdf]

# DECLARATION OF CONTRIBUTIONS TO ARTICLE

**ADMC**

| Manuscript Number: | Journal Name: | |
|---|---|---|
| CDDIS-22-3364RR | *Cell Death & Disease* | (the 'Journal') |

Proposed Title of the Contribution:

Neuron-secreted NLGN3 ameliorates ischemic brain injury via activating Gαi1/3-Akt signaling (the 'Contribution)

Author(s):

Zhi-guo Chen, Xin Shi, Xian-xian Zhang, Fang-Fang Yang, Ke-ran Li, Qi Fang, Cong Cao, Xiong-hui Chen and Ya Peng (the 'Authors')

For all *CDDis* articles, each person named as an author in the published version must be able to show he or she has contributed substantially to the article.

Authorship credit should be based on 1) substantial contributions to conception and design, acquisition of data, or analysis and interpretation of data; 2) drafting the article or revising it critically for important intellectual content; and 3) final approval of the version to be published. Authors should meet conditions 1, 2 and 3.

Any person who cannot be shown to have made a substantial contribution to the article cannot be listed as an author in the final version. The name of any person who is deemed to have made a minor contribution can, however, appear in the Acknowledgments section of the article.

Please complete the table below to indicate the contributions of all named authors to the manuscript.

| Author Full Name: | Specification of Contribution to the Manuscript: |
|---|---|
| Zhi-guo Chen | Functional assays in neuronal cells, testing signalings and cell death/apoptosis, MACO and in vivo studies, drafting the article and revising it, with final approval of the version submitted to the journal. |
| Xin Shi | Functional assays in neuronal cells, testing signalings and cell death/apoptosis, MACO and in vivo studies, drafting the article and revising it, with final approval of the version submitted to the journal. |
| Xian-xian Zhang | Functional assays in neuronal cells, testing signalings and cell death/apoptosis, MACO and in vivo studies, drafting the article and revising it, with final approval of the version submitted to the journal. |
| Fang-fang Yang | Functional assays in neuronal cells, testing signalings and cell death/apoptosis, drafting the article and revising it, with final approval of the version submitted to the journal. |
| Ke-ran Li | Functional assays in neuronal cells, testing signalings and cell death/apoptosis, drafting the article and revising it, with final approval of the version submitted to the journal. |
| Qi Fang | Data analysis and Figure organization, involved in drafting the article and revising it critically for important intellectual content, and with final approval of the version submitted to the journal. |
| Cong Cao | Data analysis and Figure organization, involved in drafting the article and revising it critically for important intellectual content, and with final approval of the version submitted to the journal. |
| Xiong-hui Chen | Data analysis and Figure organization, involved in drafting the article and revising it critically for important intellectual content, and with final approval of the version submitted to the journal. |
| Ya Peng | Data analysis and Figure organization, involved in drafting the article and revising it critically for important intellectual content, and with final approval of the version submitted to the journal. |
| | |
| | |
| | |
| | |

Please complete the table below to indicate the contributions of all named authors to the figures.

Figure 1:

Zhi-guo Chen, Xin Shi, Xian-xian Zhang, Fang-Fang Yang, Ke-ran Li, Qi Fang, Cong Cao, Xiong-hui Chen and Ya Peng

Figure 2:

Zhi-guo Chen, Xin Shi, Xian-xian Zhang, Fang-Fang Yang, Ke-ran Li, Qi Fang, Cong Cao, Xiong-hui Chen and Ya Peng

Figure 3:

Zhi-guo Chen, Xin Shi, Xian-xian Zhang, Fang-Fang Yang, Ke-ran Li, Qi Fang, Cong Cao, Xiong-hui Chen and Ya Peng

Figure 4:

Zhi-guo Chen, Xin Shi, Xian-xian Zhang, Fang-Fang Yang, Ke-ran Li, Qi Fang, Cong Cao, Xiong-hui Chen and Ya Peng

Figure 5:

Zhi-guo Chen, Xin Shi, Xian-xian Zhang, Fang-Fang Yang, Ke-ran Li, Qi Fang, Cong Cao, Xiong-hui Chen and Ya Peng

Figure 6:

Zhi-guo Chen, Xin Shi, Xian-xian Zhang, Fang-Fang Yang, Ke-ran Li, Qi Fang, Cong Cao, Xiong-hui Chen and Ya Peng

Figure 7-Figure 9

Zhi-guo Chen, Xin Shi, Xian-xian Zhang, Fang-Fang Yang, Ke-ran Li, Qi Fang, Cong Cao, Xiong-hui Chen and Ya Peng

| Signed for and on behalf of the Author(s): | Print Name: | Date: |
| --- | --- | --- |
| 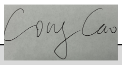 | Cong Cao | 10-12-2023 |
